# Stress gates an astrocytic energy reservoir to impair synaptic plasticity

**DOI:** 10.1038/s41467-020-15778-9

**Published:** 2020-04-24

**Authors:** Ciaran Murphy-Royal, April D. Johnston, Andrew K. J. Boyce, Blanca Diaz-Castro, Adam Institoris, Govind Peringod, Oliver Zhang, Randy F. Stout, David C. Spray, Roger J. Thompson, Baljit S. Khakh, Jaideep S. Bains, Grant R. Gordon

**Affiliations:** 1 Hotchkiss Brain Institute, Cumming School of Medicine, Department of Physiology and Pharmacology, University of Calgary, Calgary, Canada; 2 Department of Physiology, David Geffen School of Medicine, University of California Los Angeles, UCLA, Los Angeles, CA USA; 3 Department of Biomedical Sciences, New York Institute of Technology College of Osteopathic Medicine, Old Westbury, NY USA; 4 Dominick P. Purpura Department of Neuroscience, Albert Einstein College of Medicine, Bronx, NY USA

**Keywords:** Astrocyte, Stress and resilience, Long-term potentiation

## Abstract

Astrocytes support the energy demands of synaptic transmission and plasticity. Enduring changes in synaptic efficacy are highly sensitive to stress, yet whether changes to astrocyte bioenergetic control of synapses contributes to stress-impaired plasticity is unclear. Here we show in mice that stress constrains the shuttling of glucose and lactate through astrocyte networks, creating a barrier for neuronal access to an astrocytic energy reservoir in the hippocampus and neocortex, compromising long-term potentiation. Impairing astrocytic delivery of energy substrates by reducing astrocyte gap junction coupling with dominant negative connexin 43 or by disrupting lactate efflux was sufficient to mimic the effects of stress on long-term potentiation. Furthermore, direct restoration of the astrocyte lactate supply alone rescued stress-impaired synaptic plasticity, which was blocked by inhibiting neural lactate uptake. This gating of synaptic plasticity in stress by astrocytic metabolic networks indicates a broader role of astrocyte bioenergetics in determining how experience-dependent information is controlled.

## Abstract (web summary)

Enduring changes in synaptic efficacy are highly sensitive to stress. Here, the authors show that astrocytic delivery of metabolites has an important role in the stress-mediated impairment of synaptic plasticity.

## Introduction

The coordinated behavioral and physiological response to stress requires the recruitment of distinct brain pathways, release of multiple neuromodulators, and changes in peripheral hormones that promote adaptation and survival of the organism^1,2^. Acute stress also causes changes in brain function, including a well-described amnesia that accompanies intense stress^3–7^. In animal models, this requires the direct action of the circulating stress hormone corticosterone (CORT) on glucocorticoid receptors in the hippocampus^8^. Long-term potentiation (LTP) of synapses in the brain is thought to be one of the key cellular substrates for memory formation. Stress, through activation of glucocorticoid receptors inhibits LTP, but our understanding of the cellular and molecular targets of glucocorticoids following acute stress, remains largely unknown.

Glucocorticoid receptors are widely distributed throughout the brain. Their expression, however, is neither limited to neurons nor uniform across cell-types, with clear demonstrations of expression in glia and vascular endothelial cells^9^. Intriguingly, astrocytes show a seven-fold higher expression of glucocorticoid receptors compared to neurons^9^. Human postmortem brain tissue from patients afflicted with the stress-related disorder major depression, as well as animal models of stress disorders, show alterations in astrocyte structure and altered expression of proteins such as gap-junction channels, glutamate transporters, and water channels^10–15^. Many of the effects of in vivo stress on astrocyte protein synthesis and expression can be reproduced with exposure to stress hormones alone^16^. Considering that astrocytes line the cerebrovasculature with their specialized endfoot processes and express abundant receptors for glucocorticoids, these cells could be a primary target for bloodborne stress effectors. Additionally, demonstrations that astrocytes impact neuronal excitability, neurotransmitter clearance, synaptic plasticity, and the availability of bioenergetic substrates suggest these cells are positioned to link peripheral hormone changes to synaptic changes after stress^17–22^.

In the periphery, one of the primary functions of glucocorticoids is to rapidly mobilize energy stores, by stimulating gluconeogenesis from the liver. In the brain, glucose is a key energy substrate, but recent work demonstrates that lactate provided by astrocytes, which ultimately derives from free or stored glucose, is essential for the induction of LTP and memory formation in the hippocampus^17,23^. Impairments in either lactate release from astrocytes or uptake by neurons compromises LTP^17^. Upstream of the local delivery of lactate to synapses, astrocytes have broader reach by moving lactate across gap junction channels to other astrocytes^24^. This effectively creates a distributed energy reservoir that is shared among a network of astrocytes to shuttle lactate to active synapses on demand. In this hypothesis, astrocyte networks constitute an energy “grid” for neurons, whereby fuel passively moves down a concentration gradient in parallel to synaptic utilization sinks. Genetic and pharmacological manipulations that decrease gap-junction channel expression or function in astrocytes also impair LTP^25,26^. We hypothesized that corticosteroids, liberated during acute stress, decrease astrocytic network connectivity to impair the shuttling of L-lactate from astrocytes to neurons resulting in a failure of LTP expression.

Here we show that acute stress modifies astrocyte structure and function, inducing hypertrophy and decreasing gap junction coupling between cells. This stress-induced decrease in coupling effectively isolates individual astrocytes from their network, reducing their capacity to supply neurons with l-lactate, resulting in a decrease in LTP of synaptic transmission. In support of this observation, we show that genetic manipulation of astrocytic gap junction channel function alone, without altering astrocyte morphology, was sufficient to reproduce synaptic deficits. We were able to rescue the effects of acute stress on synaptic plasticity by specifically manipulating astrocytic metabolic function. We found that supplementing astrocytes with l-lactate recovered the on-demand release of l-lactate in response to synaptic stimulation, as suggested by the astrocyte-neuron lactate shuttle hypothesis. This work sheds light on a key role of astrocytes in limiting synaptic function in response to a single stressful event.

## Results

### Acute stress modifies astrocyte structure and function

**Fig. 1:**
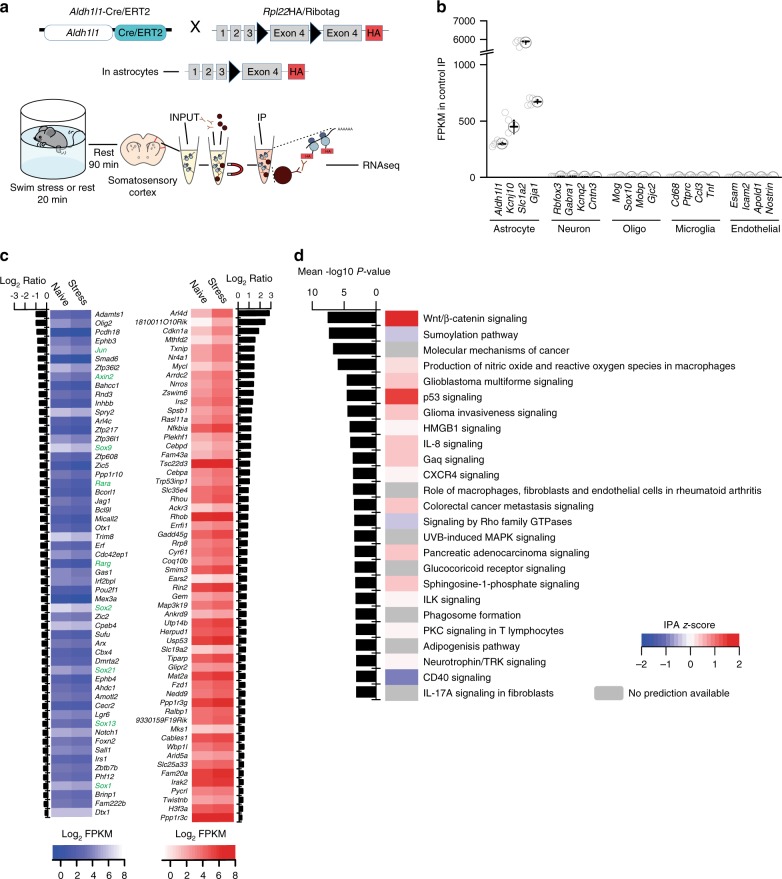
Acute stress alters astrocyte genes associated with connexins and cell growth. **a** Upper – illustration of how the knock-in mouse line used for astrocyte RNAseq experiments was generated. Lower – illustration of experimental protocol used. INPUT indicates total RNA (not specific to astrocytes). IP indicates immunoprecipitate, whereby astrocyte ribosomes were isolated. **b** Validation of RNAseq specificity using *Aldh1l1*-cre/ERT2 X RiboTag mouse, comparing the expression of several astrocyte-specific with non-specific (i.e. non-astrocytic) genes in our immunoprecipitated (IP) sample. Error bars – mean ± s.e.m. **c** Heat map of gene expression changes following stress (FDR < 0.05). Upregulated genes in red. Downregulated genes in blue. Green denotes genes involved in Wnt/B-catenin signaling pathway. **d** Top 25 IPA (Ingenuity Pathway Analysis, Qiagen) Canonical Pathways modified by a single bout of acute stress. The IPA *z*-score indicates the predicted inhibition, in blue, or activation, in red, of the pathways in accordance with the gene expression changes.

Using mice expressing an exogenous HA tag on ribosomes (Rpl22HA) specifically in astrocytes (*Aldh1l1*-cre/ERT2 x Ribotag mice) we sequenced polysomic mRNA from the neocortex of naïve mice and mice subjected to a 20-min swim stress followed by a 90-min recovery (Fig. 1a, b). Comparing the astrocyte translatome between naïve and stressed mice revealed 117 genes that were differentially affected by acute stress (Fig. 1c and Supplementary Table 1, *n* = 4 mice in each group). Detailed analysis of these data revealed a significant decrease in sox-9 (Fold change = 1.53, false discovery rate (FDR) = 0.0002; Fig. 1c; Supplementary Table 1), an astrocyte-specific transcription factor^27^ which regulates the astrocyte-enriched gap-junction channel protein connexin 30^11^. Canonical pathway analysis, which highlights the signaling cascades most strongly influenced by our manipulation, showed that stress was associated with modifications in the Wnt/β-catenin signaling (*P* = −log7.5; *z*-score = 1.89; Fig. 1d). This signaling pathway has been previously reported to be stress-sensitive, with high endogenous levels or overexpression of β-catenin conferring stress resilience^28^. Further evidence suggests that overexpression of β-catenin specifically in astrocytes is sufficient to produce anxiolytic effects^29^. Wnt/β-catenin signaling can also influence connexin 43 expression^30^, another astrocyte-specific gap-junction protein. Proper function of gap junction channels in astrocytes have been demonstrated to be pivotal for metabolic support of synaptic transmission and plasticity^31,32^. Taken together, these transcriptomic changes point toward potential alterations in astrocytic metabolic support of neuronal transmission.

Further canonical pathway analysis (Fig. 1d, highlighted modifications in pathways that have been studied in the context of cancer mechanisms e.g. 3rd most affected pathway – molecular mechanisms of cancer, indicative of cell growth and proliferation pathway upregulation. These data, in combination with the effects on transcription factors such as sox-9 that controls connexin 30 expression thereby impacting astrocyte morphology^25^, incited direct quantification of the effects of acute stress on astrocyte structure and function.

**Fig. 2:**
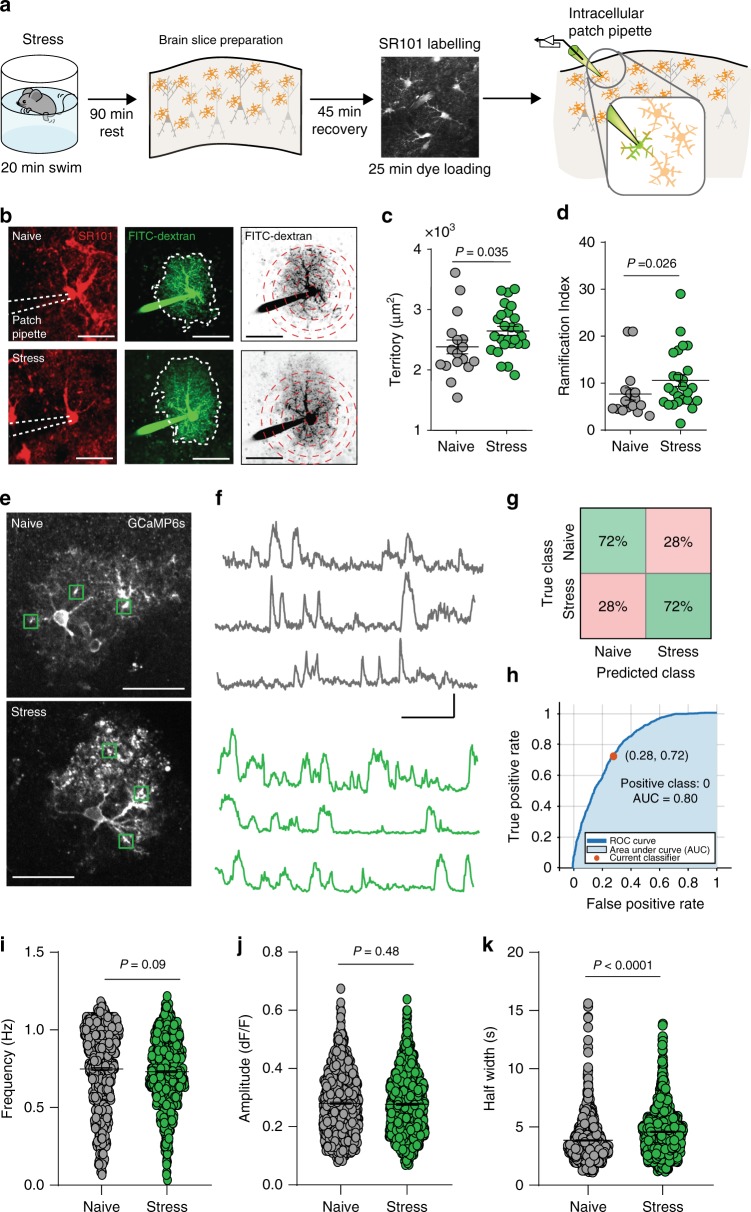
Acute stress causes astrocyte hypertrophy and prolongs spontaneous calcium events in astrocyte microdomains. **a** Timeline of experimental procedure. **b** Z-stack projection of SR101 labeled astrocytes (left), which were patched and filled with 3–5 kDa FITC-dextran (middle), for morphological analysis (right). Scale bar: 20 µm. **c** Comparison on territory sizes of individual astrocytes from naïve and stressed mice. Naïve: *n* = 5 mice, *n* = 17 cells. Stress: *n* = 6 mice, *n* = 26 cells. Error bars: mean ± s.e.m. Mann–Whitney *U*-test. **d** Comparison of astrocyte ramification in naïve and stress conditions. Error bars: mean ± s.e.m. Mann–Whitney *U*-test. **e** Representative images of GCaMP6s expression in neocortical astrocytes from naïve (top) and stressed (bottom) mice. Scale bar 35 µm. **f** Representative raw traces of microdomain calcium from naïve (top, gray) and stressed mice (bottom, green). Scale bars 2.5dF/F and 2 min. **g**, **h** Accuracy of classifier in distinguishing naïve from stressed calcium traces. **i**, **k** Scatter dot plots comparing frequency (**i**), amplitude (**j**), and half-width (**k**), of individual microdomain calcium events between naïve (*n* = 904 microdomains) and stressed (*n* = 1135 microdomains) groups. Unpaired *t*-tests.

Directed by our RNAseq data, we first assessed the effects of a single bout of acute stress on astrocyte morphology. Using patch pipettes, we filled single astrocytes in somatosensory cortical brain slices from naïve or stressed mice, with 3–5 kDa FITC-dextran (Fig. 2a, b) and then made maximum intensity projections of z-stacks through the entire astrocyte to assess the territorial domain. Astrocytes from stressed mice had larger domains than astrocytes from naïve mice (Naïve: *n* = 5 mice, *n* = 17 cells, domain = 2385 ± 113 µm^2^; Stress: *n* = 6 mice, *n* = 25 cells, domain = 2646 ± 75 µm^2^; mean ± s.e.m; Mann–Whitney *U* = 136; Fig. 2b, c) and increased ramification (Naïve: *n* = 5 mice, *n* = 17 cells, ramification index = 7.7 ± 1.3; Stress: *n* = 6 mice, *n* = 25 cells, ramification index = 10.6 ± 1.3; mean ± s.e.m; Mann-Whitney *U* = 125; Fig. 2b, d). These data demonstrate that astrocytes undergo rapid translational and structural changes following acute stress.

To determine functional adaptations to stress in single astrocytes, we investigated spontaneous astrocyte intracellular calcium activity, which relates to transmembrane fluxes, intracellular release and to mitochondrial function^33^. Using mice expressing GCaMP6s in a cre-dependent manner driven by the astrocytic promoter *Aldh1l1* (Fig. 2e, f) we observed no difference in calcium activity at the soma between naïve and stressed mice (Supplementary Fig. 2a–c). To probe stress-induced changes in astrocyte calcium at microdomains we developed a machine learning approach using a MATLAB-based artificial decoder to automatically extract quantitative metrics from calcium traces (see methods). The decoder correctly distinguished calcium traces from naïve versus stressed mice (72% accuracy Fig. 2g, h), indicating distinctive features of astrocyte calcium following a single bout of acute stress (see Supplementary Table 2 for top features used by classifier). We proceeded to quantify frequency, amplitude, and duration of individual calcium events and observed no change in the frequency (naïve: *n* = 3 mice, *n* = 24 cells, *n* = 904 microdomains, freq = 0.74 ± 0.01 Hz; stress: *n* = 3 mice, *n* = 29 cells, *n* = 1135 microdomains, freq = 0.73 ± 0.01 Hz; mean ± s.e.m; Unpaired *t*-test *t* = 1.692, df = 2037, *P* = 0.09; Fig. 2i) or amplitude (naive = 0.28 ± 0.00df/f; stress = 0.27 ± 0.03df/f; Unpaired *t*-test, *t* = 0.71, df = 2037, *P* = 0.48; Fig. 2j) of individual events. We did, however, observe an increase in the half-width of individual events (naïve = 3.85 ± 0.6 s; stress = 4.5 ± 0.06 s; mean ± s.e.m; Unpaired *t*-test, *t* = 8.6, df = 2037; *P* < 0.0001; Fig. 2k). We could also reproduce these data using a different mouse line expressing GCaMP3 under the *Slc1a3* promoter (Supplementary Fig. 2d–h).

### Stress hormones reduce functional coupling between astrocytes

**Fig. 3:**
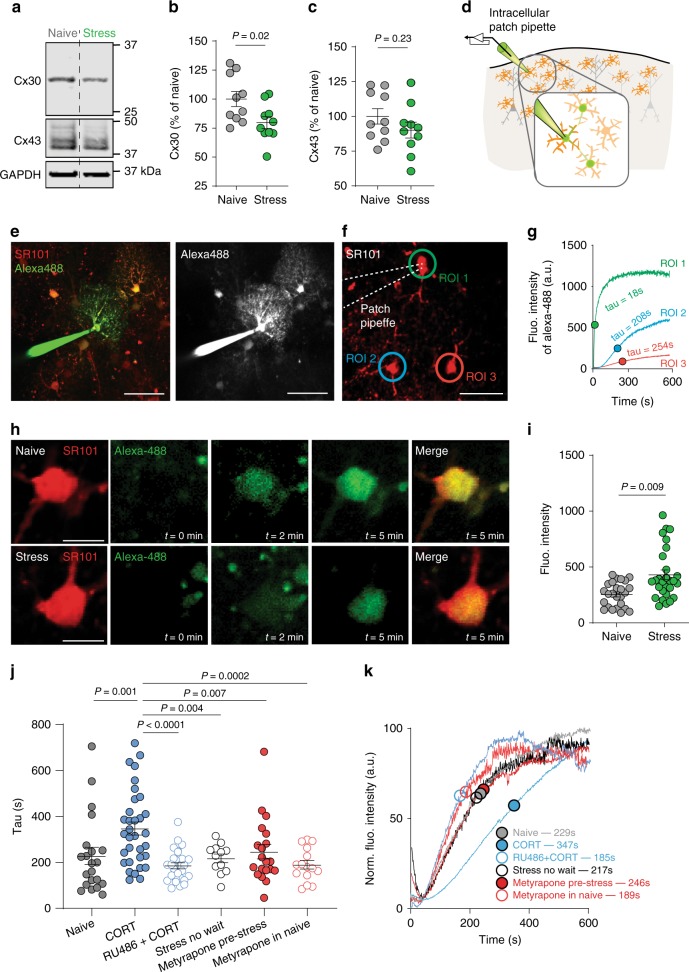
Acute stress decreases astrocytic gap junction channel expression and impairs functional coupling in a glucocorticoid-dependent manner. **a** Representative western blots comparing expression of gap junction channel proteins connexin 30 (Cx30) and 43 (Cx43) as well as the housekeeping gene GAPDH in naïve and stress conditions. **b** Summary of Cx30 expression in naïve and stress conditions. Error bars: mean ± s.e.m. *n* = 10 mice in each group. Unpaired *t*-test. **c** Summary of Cx43 expression in naïve and stress conditions. Error bars: mean ± s.e.m. *n* = 10 mice in each group. Unpaired *t*-test. **d** Schematic illustration of experiments in panels **e**–**k**. Diffusion of low molecular weight dye through astrocytic gap junctions is quantified in stress and naïve conditions. **e** Representative Z-stack projection of SR101 and Alexa-488 depicting extensive coupling of alexa-488 between astrocytes. Scale bar 35 µm. **f** SR101 was used to identify astrocytes before real-time measurement of coupling between cells. Color of ROIs correspond to **g**. Scale bar 20 µm. **g** Representative traces demonstrating kinetics of Alexa 488 filling and coupling. ROI Region of Interest. **h** Representative images depicting Alexa-488 filling in coupled astrocyte cell bodies in naïve and stress conditions. Scale bars 10 µm. **i** Tau values of filling kinetics showing slower coupling following acute stress. Naïve: *n* = 6 mice, *n* = 25 cells; Stress: *n* = 6 mice, *n* = 28 cells. Error bars: mean ± s.e.m. Unpaired *t*-test. **j** scatter dot plot depicting tau of coupled cells in naïve conditions, in CORT (100 nM; 1 h; *n* = 7 mice, *n* = 33 cells), in cocktail of RU486 + CORT (RU486 500 nM+CORT 100 nM; *n* = 3 mice, *n* = 27 cells), acute brain slice preparation directly after stress (“Stress no wait”; *n* = 5 mice, *n* = 12 cells), metyrapone pre-stress (5 mg/ml in drinking water, 24 h before swim stress; *n* = 5 mice, *n* = 20 cells), and metyrapone in naïve (5 mg/ml in drinking water, 24 h before slice experiment; *n* = 3 mice, *n* = 15 cells) Error bars: mean ± sem. One-way anova, *F* = 4.03, *P* = 0.01. **k** Mean trace of dye coupling in each condition outlined in **j** with tau value indicated.

Our transcriptome data suggested potential changes in the expression of astrocyte-enriched gap-junction channels connexin 30 and 43. Astrocytes interconnect via gap junctions, which allow the flux of small molecules across astrocyte networks, including metabolic substrates. Consistent with these insights revealed by RNA seq, we observed a decrease in connexin 30 protein expression levels (naïve = 100 ± 6.4%; stress = 79.7 ± 5.1%; *n* = 10 mice per group; mean ± s.e.m; unpaired *t*-test, *t* = 2.45, df = 18, *P* = 0.02; Fig. 3a, b). We did not, however, observe a significant change in connexin 43 protein (naïve = 100 ± 5.5%; stress = 90.1 ± 5.7%; *n* = 10 mice per group, mean ± s.e.m; unpaired *t*-test, *t* = 1.24, df = 18, *P* = 0.2; Fig. 3a, c). We also carried out a membrane enrichment protocol to examine the level of connexin 30 and 43 protein in the plasma membrane enriched fraction. We obtained results that were consistent with those observed in whole-cell lysates: a significant decrease in connexin 30, with no change in connexin 43 (Supplementary Fig. 3b–e).

To test the consequences of this decrease in gap junction channel expression in live tissue, we devised a method to examine astrocyte gap junction coupling in real time (Fig. 3d). We patch-filled individual astrocytes with Alexa-488 hydrazide (Fig. 3e; gap junction permeable) and calculated the time constant (tau) of dye flux into neighboring astrocytes with two-photon microscopy (Fig. 3f, g, Supplementary Movie 1). Importantly, the tau of the patched cell was unrelated to the tau of the coupled cell (*n* = 7 mice, *n* = 13 pairs of cells, *r*^2^ = 0.007, *P* > 0.05; Supplementary Fig. 3f, g). Additionally, the rate of dye transfer between cells was sensitive to pharmacological manipulations that increased or decreased neuronal activity (Supplementary Fig. 3h, i) as previously reported^31^. By contrast, astrocyte coupling was insensitive to extracellular glucose concentration (0, 1, 2.5, and 10 mM; Supplementary Fig. 3j). Nevertheless, to be closer to physiological concentrations^34^, we used 2.5 mM external glucose for the remainder of the experiments. Using this real-time measurement we investigated whether acute stress altered astrocyte coupling (Fig. 3h) and observed a significant increase in tau, indicating slower dye coupling between astrocytes (naïve: *n* = 6 mice, *n* = 25 cells, tau = 254.5 ± 21.9 s; stress: *n* = 6 mice, *n* = 28 cells, tau = 430.4 ± 43 s; mean ± s.e.m; Unpaired *t*-test, *t* = 3.514, df = 51, *P* = 0.0009 Fig. 3i). Considering our observation that stress induces astrocyte hypertrophy, we investigated whether the distance between astrocyte somas altered by stress. Changes in soma-soma distance could lead to an increased tau that would not be representative of changes in coupling. We report that the distance between the patched and coupled cell bodies is unaffected by stress (Supplementary Fig. 3k). The effect of stress on astrocyte coupling was transient; when measured 24 h following swim stress there was no change in the tau of coupling compared to naïve mice (Supplementary Fig. 3i).

Although we observe a stress-induced decrease in functional coupling between astrocytes, the underlying mechanism remains unexplored. Stress liberates multiple signaling molecules including catecholamines and CORT; the former acts quickly to launch the stress response while the latter has slower actions as a negative feedback signal and long-term mediator of stress. We can exploit these distinct signaling temporal windows^1^ to better understand the mechanisms involved. First, in order to limit the potential effects of glucocorticoids, we prepared acute brain slices immediately after swim stress. This provides limited time for CORT to reach an effective concentration in the brain. Under these conditions, we observed no changes in astrocyte gap junction coupling (naïve: *n* = 6 mice, *n* = 21 cells, tau = 228.6 ± 36.9 s; no wait: *n* = 5 mice, *n* = 12 cells, tau = 217.7 ± 18.5 s; mean ± s.e.m; *t* = 0.1985; *P* = 0.84; Fig. 3j, k). Next, we asked whether the increase in circulating corticosterone was required for the decrease in gap junction coupling after stress. Mice were administered the corticosterone synthesis inhibitor metyrapone through the drinking water prior to the swim stress protocol (5 mg/ml in drinking water, during 24 h pre-stress). This manipulation blocked the effects of stress on astrocyte coupling (Naïve: *n* = 6 mice, *n* = 21 cells, tau = 228.6 ± 36.9 s; Metyrapone pre-stress: *n* = 5 mice, *n* = 20 cells, tau = 246.4 ± 31.5 s; mean ± s.e.m; *t* = 0.38, *P* = 0.7; Fig. 3j, k). Importantly, we found no difference in the tau of coupling between naïve and metyrapone in naïve mice (metyrapone in naïve: *n* = 3 mice, *n* = 15 cells, tau = 189.7 ± 18.6 s, mean ± s.e.m, *P* > 0.05 compared to naïve; Fig. 3j, k) indicating that basal circulating glucocorticoids do not regulate astrocytic coupling.

Next, we asked whether CORT, in the absence of stress, was sufficient to induce changes in astrocyte coupling. We incubated brain slices from naïve animals with CORT (100 nM) for 1 h prior to imaging; this decreased astrocyte coupling (Naïve: *n* = 6 mice, *n* = 21 cells, tau = 228.6 ± 36.9 s; CORT: *n* = 7 mice, *n* = 33 cells, tau = 347.4 ± 28.6 s; mean ± s.e.m; *t* = 2.83, *P* = 0.006; Fig. 3j, k). To define the receptor responsible for the effects of CORT on astrocyte coupling, brain slices from naïve animals were pre-incubated in RU486 (a glucocorticoid receptor (GR) inhibitor; 500 nM) for 15 min before 1 h incubation in a cocktail of RU486 and CORT. In the presence of RU486, CORT had no effect on astrocyte coupling compared to naïve mice, indicating that GR activation is required for the effects of CORT on functional coupling between astrocytes (Naïve: *n* = 6 mice, *n* = 21 cells, tau = 228.6 ± 36.9 s; RU486 + CORT: *n* = 3 mice, *n* = 27 cells, tau = 185.1 ± 13.3 s; mean ± s.e.m; *P* > 0.05; Fig. 3j, k). Together these data demonstrate that glucocorticoid signaling is both necessary and sufficient for stress-induced decreases in astrocyte gap junction coupling, potentially impacting the diffusion of energetic substrates through astrocyte networks.

### Genetic disruption of astrocyte metabolic network function

**Fig. 4:**
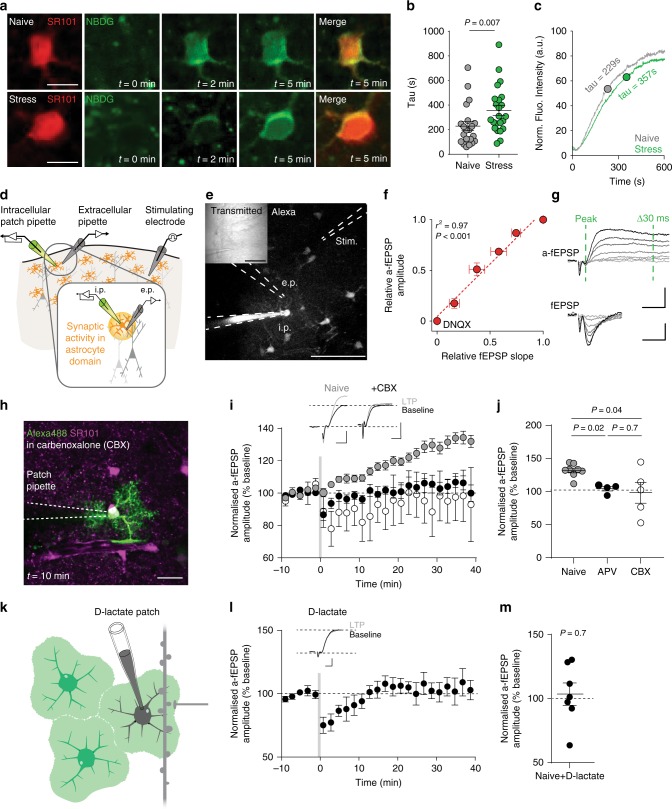
Astrocyte coupling and metabolic shuttling control LTP. **a** Representative images depicting 2-NBDG filling in coupled astrocyte cell bodies in naïve and stress conditions. Scale bars: 10 µm. **b** Tau values of filling kinetics showing slower coupling following acute stress. Naïve: *n* = 6 mice, *n* = 21 cells; Stress: *n* = 5 mice, *n* = 23 cells. Error bars: mean ± s.e.m. Mann–Whitney *U* test. **c** Mean trace of coupling in naïve and stress conditions with tau value indicated. **d** schematic diagram illustrating the placement of patch pipette, extracellular recording electrode, and stimulating electrode. **e** 2P image depicting electrode placement. Dye in the patch pipette passes between astrocytes through gap-junction channels. Inset, transmitted light image. Scale bar: 100 μm. **f** A strong linear relationship exists between the slope of the fEPSP and the amplitude of the a-fEPSP (*n* = 6 mice, *n* = 8 paired recordings). Error bars: mean ± s.e.m. Pearson’s correlation coefficient, *r* = 0.99, R2 = 0.97, *P* = 0.0003. **g** Example traces depicting changes in both a-fEPSP and fEPSP waveform with increased stimulation voltage. The amplitude of the a-fEPSP is calculated by measuring the difference (mV) between the downward peak and the point 30 ms following this peak. Scale bars for a-fEPSP: 0.5 mV, 10 ms. Scale bars for fEPSP: 0.2 mV, 10 ms. **h** Maximum intensity z-projection depicting lack of coupling between astrocytes in the presence of 100 μM carbenoxolone. Scale bar: 20 µm. **i** Normalized a-fEPSP amplitude depicting LTP impairment in the presence of the gap-junction channel blocker carbenoxolone (100 μM; *n* = 4 mice, *n* = 5 cells) and D,L-APV (50 μM; *n* = 4 mice, *n* = 4 cells). Error bars: mean ± s.e.m. Inset, example traces pre- (black) and post- (gray) LTP induction. Scale bars 0.2 mV, 20 ms. **j** Scatter dot plot depicting the extent of long-term plasticity 30–40 min following theta burst stimulation. naïve: *n* = 6 mice, *n* = 7 cells, *n* = 4 mice, *n* = 4 cells; CBX: *n* = 4 mice, *n* = 5 cells. Error bars: mean ± s.e.m. One-way ANOVA (**k**) Illustration. **l** Normalized a-fEPSP amplitude depicting the effect of patching and filling astrocytes with d-lactate on LTP in naïve animals (black). Naïve+d-lactate: *n* = 4 mice, *n* = 7 cells. Error bars: mean ± s.e.m. Inset, example average traces pre- and post-LTP in naïve and naïve+D-lactate conditions. Scale bar 0.5 mV, 5 ms in naïve and 0.25 mV, 5 ms in naïve+d-lactate. **m** Scatter dot plot indicating the extent of LTP when astrocytes were patched with d-lactate. *n* = 4 mice, *n* = 7 cells. Error bars: mean ± s.e.m. One-sample *t*-test.

The conduit provided by adjoined connexins 30 and 43 allows movement of energy substrates, such as glucose and lactate, from astrocyte to astrocyte^31^. We tested the diffusion of fluorescent glucose analog 2-NBDG through astrocyte networks and found it was no different to Alexa-488 (Supplementary Fig. 4a). We then asked whether acute stress also altered shuttling of this tagged metabolic substrate through the astrocyte network (Fig. 4a). Following swim stress, there was an increase in the time constant (tau) of filling in the secondary astrocyte, indicating slower movement of 2-NBDG between cells (naïve: *n* = 6 mice, *n* = 21 cells, tau = 228.6 ± 36.9 s; stress: *n* = 5 mice, *n* = 23 cells, tau = 356.7 ± 39.9 s; mean ± s.e.m; Mann–Whitney *U* = 128, *P* = 0.007; Fig. 4b, c). These findings indicate that acute stress decreases the shuttling of energy substrates between astrocytes.

Astrocyte connexin 30 or connexin 43 deficient animals show compromised LTP^25,26,35^. Connexin 30 is important for morphological changes that modify astrocyte fine processes infiltration of synapses^25^. The mechanistic link between astrocyte connexin 43 and LTP, however, is unresolved. One possibility is that connexin 43 is required to optimally traffic energy substrates from one astrocyte to another, ultimately towards neural energy sinks. To begin to address the role of astrocyte networks in LTP, we measured synaptic activity using classical field excitatory postsynaptic potentials (fEPSPs) combined with a method to record synaptic events through a patched astrocyte: an ‘a-fEPSP’ (Fig. 4d, e)^18,36–38^. One advantage of the latter approach is that it permits the intracellular manipulation of the patched astrocyte while simultaneously assessing changes in the strength of synapses within the domain of that astrocyte. Although this has been used effectively in the hippocampus, it has not been tested in other brain regions. We validated the use of this approach in cortical brain slices by simultaneously measuring classical fEPSPs and a-fEPSPs. We ensured that a-fEPSPs, like fEPSPs, are glutamate receptor and neuronal-activity dependent (blocked by DNQX and TTX; Fig. 4f; Supplementary Fig. 4b, c). Furthermore, simultaneous recording revealed that both methods provide comparable synaptic information (Fig. 4f, g), scaling identically with afferent electrical stimulation voltage.

To assess the functional relevance of astrocyte network integrity in neocortical plasticity in naïve mice, we first carried out experiments in the presence of the non-selective gap junction channel blocker carbenoxolone (CBX; 100 μM). CBX blocked dye flux between astrocytes (Fig. 4h, Supplementary Movie 2) and prevented LTP (naïve: *n* = 6 mice, *n* = 7 cells, LTP = 132 ± 4%; CBX: *n* = 4 mice, *n* = 5 cells, 98.2 ± 15%; mean ± s.e.m; Unpaired *t*-test, *t* = 2.59, df = 11, *P* = 0.025; Fig. 4i, j), suggesting that LTP in this brain region relies on functional astrocyte networks. Although neocortical LTP shows temporal kinetics that are distinct from those reported in other brain regions (notably, a lack of short-term potentiation), it was eliminated by D,L-APV (50 μM; *n* = 4 mice, *n* = 4 cells; LTP = 104 ± 3.5%, mean ± s.e.m), indicating it is also NMDA receptor-dependent (Fig. 4i, j). Next, we took advantage of this approach to interfere with intracellular energy substrate pathways in astrocytes, while simultaneously measuring the impact on surrounding synaptic activity. We disrupted the astrocyte to neuron lactate shuttling by patch-filling astrocytes with d-lactate (Fig. 4k). Bath application of d-lactate impairs l-lactate dependent effects on myelination, synaptic transmission, and memory^39–41^. Intracellular infusion of d-lactate, a metabolically inert but transportable monocarboxylate, should result in activity-dependent d-lactate release, competing with endogenous l-lactate for transport through monocarboxylate transporters, and shuttling to neurons. If astrocyte-derived l-lactate is required for LTP, this manipulation should impair this form of plasticity. Indeed, patch-filling astrocytes with 2 mM d-lactate completely blocked LTP in naïve mice (Naïve: *n* = 8 mice, *n* = 8 cells LTP = 132 ± 3.5% of baseline; Naïve+d-lactate: *n* = 4 mice, *n* = 7 cells, LTP = 104 ± 8% of baseline; mean ± s.e.m; *t* = 3.16, df = 13, *P* = 0.008; Fig. 4l, m). Together these observations indicate that astrocyte coupling, and astrocyte-derived energy substrates are critical for the expression of LTP in the neocortex.

### Synaptic plasticity depends upon astrocyte syncytium integrity

**Fig. 5:**
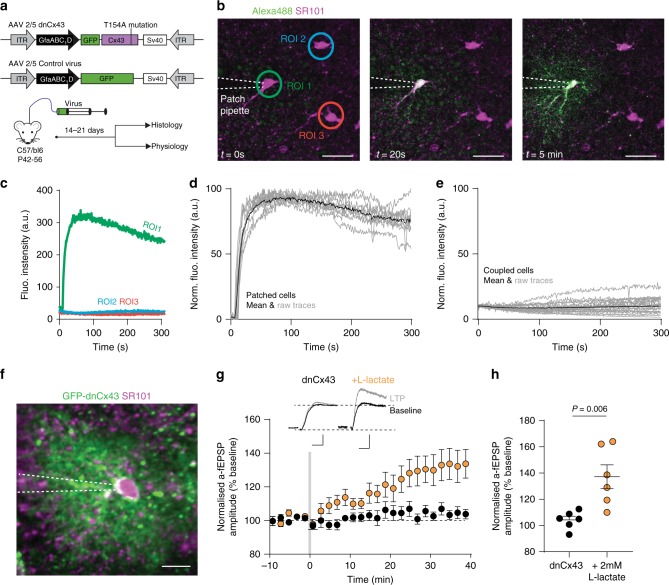
Reduction of connexin 43 gap junction channel function impairs LTP and can be rescued by overcoming a syncytial energetics deficit. **a** Diagram indicating dnCx43 construct with GFP-tag and single point mutation and control GFP virus, both under GfaABC_1_D promoter. Adult mice were injected before histology and physiological recordings. **b** Representative images of the effect of dnCx43 expression on astrocyte coupling at *t* = 0 s (before patching), *t* = 20 s following whole-cell patch-clamp where dye is in patched astrocyte, and at *t* = 5 min where dye remains in patched cell and has not spread to nearby astrocytes. Scale bar 35 µm. **c** Raw data showing alexa-488 fluorescence intensity changes in astrocytes from **b**. **d** Normalized fluorescence intensity of patched cells expressing dnCx43. Black trace is mean, gray traces are raw data from 10 patched astrocytes. **e** Normalized fluorescence intensity of coupled cells expressing dnCx43. Black trace is mean, gray traces are raw data from 21 coupled astrocytes. **f** Two-photon average intensity z-projection of single dnCx43-expressing astrocyte co-labeled with SR101. Scale bar 15 µm. **g** Normalized a-fEPSP amplitude demonstrating effects of dnCx43 (black) on LTP induced by 10 s theta burst stim (gray bar), which was rescued by supplementing astrocytes with 2 mM l-lactate (orange). dnCx43: *n* = 5 mice, *n* = 6 cells. dnCx43 + l-lactate: *n* = 5 mice, *n* = 6 cells. Error bars: mean ± s.e.m. Unpaired *t*-test. Inset, example traces pre- (black) and post- (gray) LTP induction. Scale bars 0.5 mV, 10 ms. **h** Scatter dot plot depicting the extent of long-term plasticity 30–40 min following theta burst stimulation (data from **g**). Error bars: mean ± s.e.m. Unpaired *t*-test.

Our observations are consistent with the hypothesis that stress, through a glucocorticoid-dependent reduction in astrocytic gap-junction coupling, diminishes the capacity of astrocytes to maintain the supply of energy substrates to synapses, consequently impairing LTP. Despite the clear effect of CBX on astrocyte coupling, off-target effects of CBX on NMDA receptor function have been reported^42^. Thus, we asked whether disrupting cortical astrocytic metabolic networks by targeting connexin 43 directly would impact LTP. To do this, we developed a viral approach to express a dominant-negative connexin 43 (dnCx43) driven by a 600-bp fragment of the GFAP promoter (GfaABC_1_D) in astrocytes (Fig. 5a). The dnCx43 construct was produced by tethering a GFP to the N-terminus of connexin 43, with an additional dominant-negative point mutation (from threonine to alanine) at the 154th amino acid position. A similar dominant negative approach has been used to eliminate connexin 43 gap junction communication in cultured cell lines^43^. This combination effectively blocked gap junction intercellular communication, and putative hemichannels, by eliminating conductance through connexin 43 channels that contain both endogenous connexin 43 and dnCx43, while allowing formation of inactive gap junction adhesion structures. This dnCx43 approach has the advantage of minimally interfering with non-channel based activities that are carried out by gap junctions^44^. We carried out extensive characterization of the effects of dnCx43 expression and report impaired dye coupling in cultured cells (Supplementary Fig. 5a–k), reduced electrical coupling between astrocytes in brain slices (Supplementary Fig. 5l–n), decreased size of astrocyte syncytial networks (Supplementary Fig. 5o, p), and small but significant changes to resting membrane potential without affecting whole-cell conductance (Supplementary Fig. 5q–u). Expression of dnCx43 did not, however, impact cell morphology (Supplementary Fig. 5v, w). The real-time dye flux assay between astrocytes revealed an almost complete occlusion of coupling between neighboring astrocytes in the neocortex expressing dnCx43 (Fig. 5b–e).

This viral approach enabled us to define the importance of astrocytic metabolic networks on cortical plasticity, without impacting astrocyte morphology that could alter physical interactions between astrocyte processes and synaptic elements. Patching individual dnCx43-expressing astrocytes, we used the a-fEPSP technique to record the impact on synapses in the domain of a single astrocyte. We failed to induce LTP in brains slices from mice transfected with the dnCx43 construct (dnCx43 *n* = 5 mice, *n* = 6 cells, LTP = 104.3 ± 2.7%, mean ± s.e.m.; Fig. 5f–h). This was not due to viral expression alone (Supplementary Fig. 5x, y). Having demonstrated that decreased coupling between astrocytes resulted in reduced shuttling of metabolic substrates between cells, we hypothesized that overcoming astrocytic metabolic network deficits could rescue LTP impairments. Remarkably, filling a single dnCx43-expressing astrocyte with 2 mM l-lactate rescued LTP (dnCx43 + l-lactate *n* = 5 mice, *n* = 6 cells, LTP = 137.2 ± 9.1%; mean ± s.e.m.; Unpaired *t*-test, *t* = 3.48, df = 10, *P* = 0.006; Fig. 5f–h). These data revealed that individual astrocytes, isolated from their network, are incapable of meeting the energy demands required for synaptic plasticity, highlighting the vital role of astrocytic networks acting as a synaptic energy reservoir.

### Astrocyte to neuron lactate shuttle is impaired by stress

**Fig. 6:**
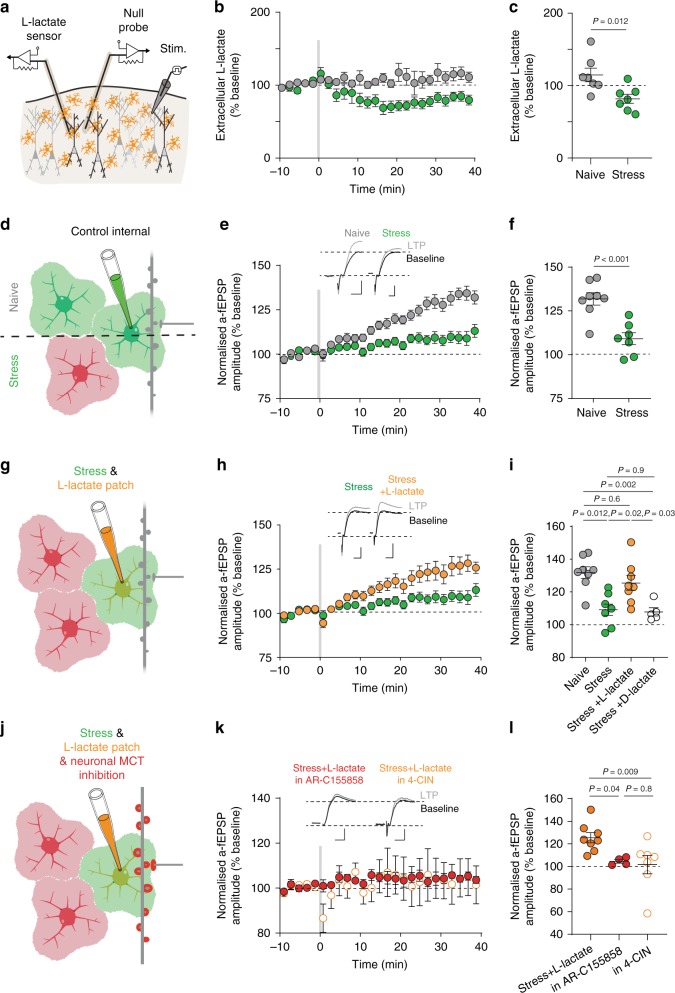
l-lactate delivery through astrocytes rescues stress-induced impairment of LTP. **a** Schematic representation of measuring extracellular l-lactate concentration using enzymatic probes. One probe was coated in an enzymatic layer specific to l-lactate generating l-lactate specific signals, one ‘null probe’ did not have the enzymatic layer and was used to account for non-specific probe readings, and a monopolar stimulating electrode placed nearby. **b** Normalized extracellular l-lactate concentration in naïve (gray) and stress (green) conditions. Gray bar depicts theta burst stim. Error bars: mean ± s.e.m. **c** Scatter dot plot extracellular l-lactate levels at 30–40 min post-stim. Error bars: mean ± s.e.m. Unpaired *t*-test. **d** Schematic representation of experiments in **e** and **f**, recording a-fEPSPs from cortical astrocytes in naïve (top) and stress conditions (bottom), where coupling is reduced. **e** LTP is impaired following acute swim stress. mean ± s.e.m. Inset, example a-fEPSP traces pre- (black) and post-LTP (gray) induction. Scale bars: 0.5 mV, 10 ms. **f** Comparison of LTP between naïve and stressed mice. Error bars: mean ± s.e.m. Unpaired *t*-test. **g** Diagram representing following experiments (**h**, **i**). Astrocytes from stressed mice were patched with l-lactate and the effect on synaptic activity in this cell’s domain was recorded. **h** Normalized a-fEPSP amplitude depicting extent of recovery of LTP with supplementation of l-lactate into the patched astrocyte. Error bars: mean ± s.e.m. Inset, example a-fEPSP traces pre- and post-LTP induction in naïve (scale: 0.5 mV,10 ms), stress (scale: 0.5 mV,10 ms), stress+l-lactate (scale: 1.0 mV,10 ms). **i** Scatter dot plot comparing the extent of LTP in each condition (Naïve: *n* = 8 mice, *n* = 8 cells; Stress: *n* = 8 mice, *n* = 8 cells; Stress+l-lactate:*n* = 6 mice, *n* = 7 cells; Stress+d-lactate:*n* = 5 mice, *n* = 5 cells; mean ± s.e.m.). Error bars: mean ± s.e.m. One-way Anova, *F*(3,24) = 9.55, *P* = 0.0002. **j** Schematic diagram representing following experiments (**k**, **l**). Astrocytes were patched with l-lactate and the effect on synaptic activity in this cell’s domain was recorded, in the presence of the monocarboxylate transporter inhibitors AR-C155858 (1 µM) or 4-CIN (100 µM) blocking neuronal l-lactate uptake. **k** Normalized a-fEPSP amplitude depicting lack of recovery of LTP with supplementation of l-lactate into the patched astrocyte when blocking neuronal l-lactate uptake. Error bars: mean ± s.e.m. Inset, representative a-fEPSP traces pre- and post-LTP induction. scale: 1 mV,10 ms. **l** Scatter dot plot comparing LTP amplitude between Stress+l-lactate and Stress+l-lactate in the presence of AR-C155858 or 4-CIN. Error bars: mean ± s.e.m. One-way ANOVA, *F* = 4.91, *t* = 0.02.

We have shown that creating an artificial energy reservoir through delivery of l-lactate through the patch pipette can rescue the effects of impaired astrocyte coupling on synaptic plasticity. Indeed, stress reduces energy substrate shuttling between astrocytes but whether this disrupts activity-dependent release of energy substrates by astrocytes is unknown. To determine the effects of stress on lactate shuttling, we used enzymatic biosensors to quantify l-lactate levels in the extracellular space in neocortical tissue from naïve and stressed mice. Using this approach, we found that basal l-lactate concentration was not different in brain slices from naïve and stressed mice (Supplementary Fig. 6a). Following theta burst stimulation, we observed no change in extracellular l-lactate levels in slices from naïve animals (*n* = 3 mice, *n* = 7 brain slices, LAC post TBS = 114 ± 9% of baseline, mean ± s.e.m.; Fig. 6b, c), indicating that the system is able to maintain adequate levels of extracellular lactate after intense synaptic activity. By contrast, the same protocol delivered to slices from stressed animals resulted in a decrease in tissue lactate (*n* = 3 mice, *n* = 7 brain slices, LAC post TBS = 81 ± 6%, mean ± s.e.m.; Unpaired *t*-test, *t* = 2.94, df = 12, *P* = 0.012; one-sample *t*-test to compare to baseline; naïve: *t* = 1.59, df = 6, *P* = 0.16; stress: *t* = 2.88, df = 6, *P* = 0.027; Fig. 6b, c). Importantly, we observed no difference in the initial stim-induced release of l-lactate between naïve and stressed mice (naïve - 130 ± 12.8%, stress - 124.5 ± 7.3% of baseline; Supplementary Fig. 6b). This suggests that while astrocytes retain the capacity to release l-lactate in response to stimulation, sustained l-lactate release is compromised after stress.

Considering that acute stress leads to astrocytic hypertrophy, altered calcium activity, decreased intercellular astrocyte coupling, and finally a decay in activity-dependent l-lactate shuttling, we wondered whether these astrocyte-specific modifications have functional consequences for synapses. We hypothesized that the effects of stress on astrocyte function could underlie commonly observed LTP impairments associated with stress in various brain regions^45,46^. Using the astrocyte-fEPSPs approach, we found a significant impairment of LTP at neocortical synapses (naïve: *n* = 6 mice, *n* = 8 cells, LTP = 132 ± 4%; stress: *n* = 6 mice, *n* = 7 cells, LTP = 109 ± 3%, mean ± s.e.m.; *t* = 4.577, df = 13. *P* < 0.001; Fig. 6d–f, and Supplementary Fig. 3e, f). We corroborated these results by verifying that the extent of the stress-impaired LTP was not influenced by the astrocyte-patch technique using classical field recordings (Supplementary Fig. 6a–c).

To link astrocytic metabolic dysfunction with decreased LTP, we predicted a rescue of stress-impaired plasticity by creating an artificial energy reserve within astrocytes. In slices from stressed mice, we supplied single astrocytes with l-lactate (2 mM; Fig. 6g) through a patch pipette, effectively creating an energy reservoir to overcome astrocyte network dysfunction. This manipulation resulted in the recovery of LTP to non-stressed levels (stress: *n* = 7 mice, *n* = 7 cells, LTP = 109 ± 3%; stress+l-lactate: *n* = 6 mice, *n* = 7 cells, LTP = 126 ± 5%, mean ± s.e.m.; *q* = 4.414, df = 24, *P* = 0.02; Fig. 6h, i). By contrast, including d-lactate in the astrocyte patch pipette failed to rescue stress-impaired LTP, reflecting the importance of l-lactate derived ATP (Stress: *n* = 7 mice, *n* = 7 cells, LTP = 109 ± 3%; Stress+d-lactate: *n* = 5 mice, *n* = 5 cells, LTP = 108 ± 3%, mean ± s.e.m.; q = 0.32, df = 24, *P* = 0.99; Fig. 6i). Finally, to determine whether l-lactate needs to be released and taken up by neurons, we blocked l-lactate trafficking by inhibiting monocarboxylate transporters using AR-C155858 (blocking MCT1 and 2, with no activity at astrocytic MCT4^47^; 1 µM), or 4-CIN (effective on MCT2 at 100 µM^48^; Fig. 6j). Both drugs independently abolished the rescue of LTP by l-lactate (stress+l-lactate in AR-C155858: *n* = 4 mice, *n* = 4 cells, LTP = 104.5 ± 1.7%; stress+l-lactate in 4-CIN: *n* = 6 mice, *n* = 6 cells, LTP = 102  ±  8%, mean ± s.e.m.; One-way ANOVA, F = 4.91, *P* = 0.02; Fig. 6k, l), indicating that the rescue effect of l-lactate in these conditions is likely due to metabolic consumption rather than direct action on receptors as reported elsewhere^49,50^.

**Fig. 7:**
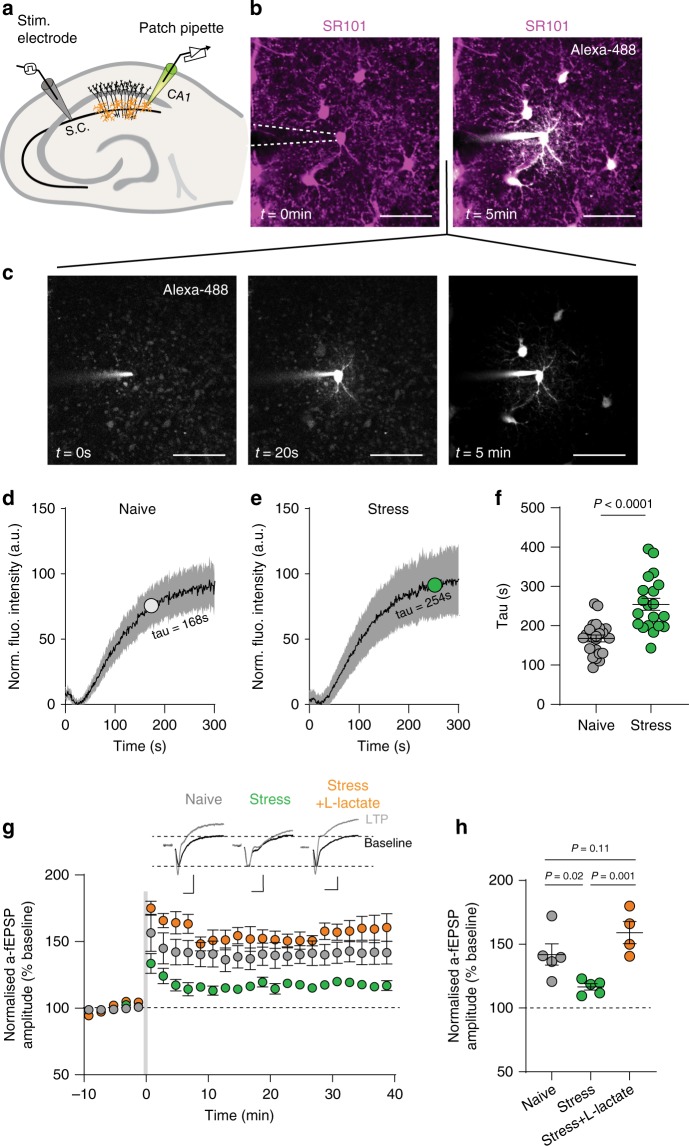
l-lactate delivery through astrocytes rescues stress-induced impairment of LTP in the hippocampus. **a** Schematic representation of subsequent patch-clamp experiments in CA1 region of ventral hippocampus (s.c. denotes schaffer collateral). **b** Representative images depicting a dye flux through astrocyte network in ventral hippocampal slices, before patching (left) and 5 min after whole-cell patch (right). Scale bar 50 µm. **c** Series of images from **b** depicting progression of dye flux through gap junction connected astrocytes at 0 s, 20 s, and 5 min. **d** Mean trace of coupling in naïve group with tau value indicated. Error indicates standard deviation. *n* = 23 cells**. e** Mean trace of coupling in stress group with tau value indicated. Error indicates standard deviation. *n* = 20 cells. **f** Scatter dot plot quantifying coupling between hippocampal astrocytes in naïve and stress conditions. Naïve: *n* = 23 cells, stress: *n* = 20 cells. Error bars: mean ± s.e.m. **g** Time series comparing hippocampal LTP in naïve, stress, and stress+l-lactate conditions, measured through astrocyte-patch technique. High-frequency stimulation (HFS) indicated by gray bar. Naïve: *n* = 3 mice, *n* = 5 cells; Stress: *n* = 3 mice, *n* = 5 cells; Stress+l-lactate: *n* = 3 mice, *n* = 4 cells. Error bars: mean ± s.e.m. Inset, representative a-fEPSP traces pre- and post-LTP induction. Scale bars: 0.5 mV,10 ms. **h** Scatter dot plot comparing amplitude of potentiation at 30–40 min post HFS from **g**. Error bars: mean ± s.e.m. One-way ANOVA, *F* = 9.46, *P* = 0.0041.

Finally, we asked whether our results were neocortex-specific or conserved across different brain structures. We chose to carry out key experiments in the hippocampus, a brain region that has been shown to be robustly affected by stress and associated with synaptic plasticity and memory impairment^45,46^. We report that, consistent with our observations in the neocortex, stress reduces functional coupling between hippocampal astrocytes (Naïve: *n* = 3 mice, *n* = 23 cells, tau = 168 ± 8 s; stress: *n* = 3 mice, *n* = 20 cells, tau = 254 ± 15 s, mean ± s.e.m.; unpaired *t*-test, *t* = 5.12, df = 41, *P* < 0.001; Fig. 7a–f). Considering that the reduction in functional coupling in neocortical astrocytes was associated with impairment in synaptic plasticity, we replicated this experiment, patching astrocytes and recording a-fEPSPs in CA1 region of ventral hippocampus. Using a-fEPSP approach to record synaptic activity in single astrocyte domains, we observed classical hallmarks of hippocampal plasticity, such as STP (Fig. 7g). Again, we observed that a single bout of acute stress decreased long-term potentiation (Naïve: *n* = 3 mice, *n* = 5 cells, LTP = 142 ± 8.3%; Stress: *n* = 3 mice, *n* = 5 cells, LTP = 116 ± 2.5%, mean ± s.e.m.; Fig. 7g, h). Furthermore, hippocampal LTP was rescued by patch-filling astrocytes with 2 mM l-lactate as in the neocortex (Stress + l-lactate: *n* = 3 mice, *n* = 4 cells, LTP = 159 ± 8.7%, mean ± s.e.m.; One-way ANOVA, *F* = 9.46, *P* = 0.0041; Fig. 7g, h). These data demonstrate that stress restricts synaptic plasticity by impairing the capacity of astrocytes to maintain synaptic energy homeostasis in both the neocortex and hippocampus.

## Discussion

In this study we describe a mechanism by which stress, through discrete changes to astrocytic metabolic networks, limits neuronal access to an astrocytic energy reservoir thereby impairing long-term plasticity. We describe an essential role of the astrocyte syncytium, demonstrating that connectivity between astrocytes serves as a distributed energy source, capable of fueling neurons under conditions of high-energy demand, such as high-frequency neuronal stimulation commonly used to induce LTP. This suggests that although each astrocyte can make and deliver its own energy molecules to local synapses, mobilization of intracellular astrocytic energy stores is necessary to support “hot spots” of synaptic activity. Energy demands from individual synapses undergoing plasticity, or other energy intensive processes, are spatially and temporally separated; astrocytes through their extensive coupling coordinate the delivery of energy substrates on this synaptic spatiotemporal scale. Analogous to an energy grid distributing electricity through a network, astrocyte metabolic networks increase the reliability of matching energy supply with demand, decreasing the burden of activity-dependent energy production by any one individual cell.

We report that acute stress affects distinct facets of astrocyte morphology, calcium signaling, and intercellular coupling. Whether a direct link between these aspects exists remains unresolved, nevertheless all three point towards potential metabolic dysfunction. First, stress-induced changes microdomain calcium signaling could relate at least partly to altered mitochondrial function. Some, but not all, astrocyte microdomain calcium events localize to mitochondria^51,52^, and may depend on calcium efflux from opening of the permeability transition pore (mPTP)^53^ as well as intracellular release and transmembrane fluxes. Inhibition of the mPTP^53^ or impairment of mitochondrial mobility^51,52^ lead to changes in the duration of microdomain calcium events, similar to our finding in stress. Based on these previous studies we speculate that stress may impair mitochondrial function and/or positioning in astrocytic fine processes. This could have implications for ATP-dependent mechanisms in perisynaptic astrocyte processes, such as Na^+^/K^+^-ATPase pumps and interdependent processes such as glutamate uptake which relies on robust sodium concentration gradients. Second, stress-induced astrocyte hypertrophy could physically uncouple astrocyte gap junction channels. Rapid alterations in astrocyte morphology could also reduce reflexive gap junction channel formation between distinct processes in the same astrocyte. Considering that we observed modest changes is connexin protein expression following stress, associated with a strong functional phenotype assayed with dye flux experiments, it is possible that stress-induced astrocytic hypertrophy ruptures existing gap junction channels, thereby increasing the number of hemichannels without modifying protein expression. This is consistent with previous work suggesting that stress increases hemichannel expression in hippocampal astrocytes^54^. In this manner, stress-induced morphological change has the capacity to directly impact cellular metabolism by decreasing energy substrate shuttling in the astrocyte syncytium, shifting the burden of energy supply from the network onto individual cells which are unable to meet the demand of LTP. Another possibility includes rapid post-translational modifications of connexin 43 gap junction channels that have been demonstrated using the synthetic glucocorticoid receptor agonist dexamethasone, leading to rapid channel closure that does not require removal of the junctional structure^55^. This could be another means by which CORT, through activation of glucocorticoid receptors, is altering functional coupling in the context of stress.

Our observations suggest that stress-induced synaptic plasticity impairment is mediated by a synaptic energy deficit, rather than changes in neuronal or glia-neuron signaling. The evidence we provide includes pharmacological inhibition of monocarboxylate transporters. AR-C155858, which blocks MCT1 and MCT2 with no activity at the astrocyte-specific MCT4, as well as low-concentration of 4-CIN which has been reported to reduce neuronal uptake of l-lactate, blocked the l-lactate mediated rescue of synaptic plasticity. Recent demonstration of the capacity of MCT4 to release abundant l-lactate under physiological conditions^56^ further supports our hypothesis that neuronal uptake of l-lactate is necessary for synaptic potentiation^17^. Furthermore, our use of enzymatic l-lactate sensors reveals that while the immediate stimulation-induced increase in extracellular l-lactate is unaffected by stress, a stress-induced decrease in extracellular l-lactate that is not replenished from energy stores becomes apparent at later time points. Indeed, previous work demonstrated that the effects of CORT on synaptic function could be rescued by extracellular l-lactate supplementation^57^. Importantly, our data suggest that l-lactate release is mediated by MCTs rather than connexin hemichannels, as l-lactate was able to rescue LTP when dnCx43 was expressed in astrocytes, blocking gap junction coupling and presumed hemichannel function.

The effects of stress on astrocyte function appears to be conserved across the neocortex and hippocampus, regions which are tightly linked, involved in memory formation, and strongly impacted by stress. Future studies are required to investigate whether this stress-induced synaptic phenomenon occurs in other cortical or subcortical brain regions, to test for potential regional heterogeneity in the astrocytic response to stress, as has been observed outside of this context^58,59^. Indeed, this seemingly deleterious mechanism (reducing the capacity of synapses to undergo plasticity in the cortex and hippocampus) may favor energy supply and plasticity in other brain regions necessary for threat assessment and homeostatic regulation (e.g. amygdala, hypothalamus). Preserving the astrocyte-neuron lactate shuttle in survival circuits may be one means by which the central stress response may induce the appropriate behavioral response and improve the chance of survival.

Our findings extend previous work implicating the astrocyte-neuron lactate shuttle in regulating synaptic plasticity^17^, demonstrating that gating of this system underlies stress-induced plasticity impairment. Astrocyte-derived L-lactate is critical for the maintenance of neuronal resting membrane potential^60,61^, the central regulation of blood glucose levels^62^, learning and memory in vivo^17,23,63,64^, decision making^65^, regulation of the sleep-wake cycle^32^, and here we show it is essential for controlling synaptic plasticity following stress. The effects of stress on astrocytes that we describe here, may be another example by which astrocytes respond to the external environment, guiding neuronal and brain circuit activity towards an appropriate response. These observations provide insights for understanding mechanisms of cognitive decline and memory impairment associated with stress. We propose that restoration of astrocytic metabolic function may prove useful in a variety of stress-related disorders, in which astrocyte dysfunction has been reported^66^. Rather than directly tuning neuronal excitability, which is the most common therapeutic strategy to manage stress disorders, manipulation of astrocyte metabolic function could ensure that neuronal energy consumption is matched with supply in an activity-dependent manner.

## Methods

### Animals

All animal protocols were approved by the University of Calgary Animal Care and Use Committee and were in accordance with the NIH Guide for the Care and Use of Laboratory Animals and were approved by the Chancellor’s Animal Research Committee at the University of California, Los Angeles. Both male and female C57BL6J mice were used in the present study (p25-70) with ad libitum access to food and water. Mice were housed on a 12 h:12 h light:dark cycle (lights on at 6:00 a.m.) in whole litters before use. Animals were single house 24 h before experiment. The acute stress protocol consisted of 20 min swimming in a beaker of water, before placing the mouse back in the cage for 90 min. Following 90-min recovery period animals were killed, and brain slices were prepared for imaging and electrophysiological recordings. In metyrapone experiments, drinking water was supplemented with metyrapone (adrenal steroid synthesis inhibitor; 5 mg/ml) for 24 h.

### Acute brain slice preparation

Acute coronal slices of the neocortex from male C57BL6J mice were prepared. Animals were anaesthetized with isoflurane before decapitation, and their brains were quickly and carefully removed. The brains were placed into a beaker of ice-cold slicing solution and continuously bubbled with carbogen (95% O_2_, 5% CO_2_). Slicing solution contained (in mM): 119.9 *N*-methyl-d-glucamine, 2.5 KCL, 25 NaHCO_3_, 1.0 CaCl_2_-2H_2_O, 6.9 MgCL_2_-6H_2_O, 1.4 NaH_2_PO_4_-H_2_O, and 20 glucose. Brain slices were cut on a vibratome (Leica) and placed in a recovery chamber containing carbogen bubbled aCSF for 45 min at 35 °C, following this recovery period brain slices were kept and room temperature. ACSF contained the following (in mM): 2.5 glucose, 136.9 NaCl, 2.5 KCl, 25 NaHCO_3_, 1.3 CaCl_2_, 1.2 MgCl_2_, and 1.25 NaH_2_PO_4_.

### Sulforhodamine 101 loading

Prior to imaging slices were loaded with the astrocyte marker Sulforhodamine 101 (SR101), 20 μM for 15 min at room temperature, which has been shown to avoid potential deleterious effects of SR101 on brain activity^67^. Slices were subsequently returned to recovery chamber containing aCSF for a minimum of 30 min before imaging.

### Two-photon imaging

Fluorescence imaging was performed on a custom two-photon laser-scanning microscope, optimized for acute brain slices and patch-clamp electrophysiology^68^. The microscope was equipped with a Ti:Sapph laser (Ultra II, Coherent), a green bandpass emission filter (525-40 nm), an orange/red bandpass emission filter (605–70 nm), and associated photomultiplier tubes (GaAsP, Hamamatsu). For coupling experiments, time-series images were collected using bidirectional scanning (512 × 512 pixels at 1 Hz) at a single focal plane incorporating the patched astrocyte and the cell body of at least one other astrocyte. For simultaneous imaging of Alexa-488/2-NBDG and SR101 the laser was tuned to 850 nm. To investigate the effects of neuronal activity on astrocyte coupling slices were placed in either Tetrodotoxin (TTX; 1 µM; Tocris) or 4-aminopyridine (4-AP; 50 µM; Sigma-Aldrich), for a least 20 min in the imaging chamber before commencing coupling experiments. In CORT experiments, slices were bathed in corticosterone (100 nM; Sigma-Aldrich) for 1 h or a cocktail of corticosterone and RU486 (Mifepristone; 500 nM; Sigma-Aldrich) then moved to imaging chamber for quantification of dye coupling. All images were acquired using scanimage v4.2 (vidrio technologies).

For analysis of astrocytic ramification, we used Sholl’s concentric circles technique in imageJ. Circles at 5 μm intervals were drawn around each astrocyte. The number of intersections of astrocytic processes with each circle was quantified. The longest primary process was measured by tracing the process with the ImageJ length measuring tool. Ramification index was calculated by dividing the maximum number of intersections (processes) by the number of primary processes.

### Calcium imaging and analysis

Imaging was carried out, as above, on knock-in cre-lox mice expressing GCaMP6s (Ai96, Jax 024106) under the *Aldh1l1* promoter (*Aldh1l1*-CreERT2, Jax 029655), and GCaMP3 (Jax 028764) under the astrocyte-specific *Slc1a3* promoter (Jax 012586;GLAST-CreERT x LSL-GCaMP3), exciting at 940 nm. Cortical brain slices were prepared as described above. Time series images, to assess fluctuations in intracellular astrocyte calcium, were acquired at a single focal plane using bidirectional scanning (512 pixels^2^ at 1 Hz frame rate). Individual calcium microdomains were identified and analyzed using the GECIquant plugin^69^ for ImageJ in combination with either Mini Analysis (Justin Lee, Synapsoft) for GCamP3 analysis and quantification of individual events or using MATLAB and /or classifier in MATLAB.

For individual event detection and analysis in MATLAB we developed an algorithm to profile each microdomain’s raw Ca^2+^ activity trace using the ‘findpeaks’ function in MATLAB. Specifically, we extracted the amplitude, location, full-width at half-maximum and prominence of each local maximum in the time-series trace using the following input parameters consistently across all microdomains without any pre-processing: ‘MinPeakHeight’ = 10% trimmed mean of trace; ‘MinPeakProminence’ = 20 (average peak prominence across all microdomains); ‘Threshold’ = 0; ‘MinPeakDistance’ = 5 (to extract peaks separated by at least 500 ms); ‘MinPeakWidth’ = 1 (to extract peaks of at least 100 ms duration).

For machine-learning based signal classification we used a MATLAB-based massive feature extraction framework to automatically extract quantitative metrics from the Ca^2+^ activity traces and subsequently trained a Support Vector Machine with a radial basis function kernel (SVM-RBF) in MATLAB using 5-fold cross-validation. Briefly, we aggregated and anonymized microdomain time-traces, and created a labeled raw data matrix with class labels representing stress condition (Naïve, Stressed); we extracted 7500+ features from each time trace using the HCSTA framework^70^ and used the t-distributed stochastic neighbor embedding (t-SNE) algorithm to visualize non-linear clustering in a lower-dimensional space; we utilized the Classification Learner app in MATLAB to train a Medium Gaussian SVM model (Box constraint level = 1; Kernel scale mode = Manual; Multiclass method: One-vs-One; Standardize data = ‘Yes’; PCA: Disabled) with 5-fold cross-validation for each case, and evaluated the quality of the models using standard metrics (confusion matrix, area under the receiver operating characteristic curve).

### Electrophysiological recording and analysis

Astrocytes targeted for patch-clamp were identified by SR101 staining and located at a depth of 20-50 µm from the surface of the slice. Astrocytes were patched under 2 photon-fluorescence, with the patch pipette containing a green fluorophore (Alexa-488 or 2-NBDG; Thermo-Fisher) in the internal solution. As a base, internal solutions contained the following (in mM): 108 K-gluconate, 2 MgCl_2_, 8 Na-gluconate, 8 KCl, 1 K-EGTA, 4 K-ATP, 0.3 Na-GTP, and 10 HEPES. Astrocytes were identified using multiple criteria including SR101 labeling, low input resistance, a linear current–voltage relationship, and extensive dye transfer between cells via gap-junction channels. To record field excitatory postsynaptic potentials (fEPSPs) through the astrocyte (a-fEPSP), cells were recorded in current-clamp mode. For extracellular field recordings, which were carried out to verify that our measurement of the a-fEPSP accurately represents classical fEPSPs, a pipette filled with aCSF was placed in the synaptic domain of the patched astrocyte (to sample the same synapses). A monopolar aCSF-filled electrode was placed (~100 µm) from the patched astrocyte and used to evoke EPSPs. The theta burst stimulation (TBS) lasted 10s consisting of 4 pulses at 100 Hz repeated at 5 Hz intervals. Signals were amplified using a Multiclamp 700B amplifier (Molecular Devices) and digitized using the Digidata 1440 (Molecular Devices). Data were recorded (pClamp 10.2, Molecular Devices) for offline analysis. Evoked a-fEPSP amplitude was calculated as the difference in mV between the peak amplitude of the downward deflection and the amplitude of the a-fEPSP 30 ms following this peak (as in *24*). The magnitude of LTP was calculated as the average amplitude of a-fEPSPs 30–40 min following LTP induction. In certain experiments either Na-L-Lactate or Na-D-Lactate (2 mM; Sigma-Aldrich) was added to the internal solution, and/or with 4-CIN (α-Cyano-4-hydroxycinnamic acid; 100uM; Sigma-Aldrich) or AR-C155858 (1uM; Tocris) in the bath (extracellular solution). All electrophysiological experiments were carried out at room temperature.

### Western blot

Stressed and naïve animals were killed, and neocortical tissue was rapidly extracted. Cortices were pooled for each animal and lysed using a Dounce homogenizer in Tris-based lysis buffer (150 mM NaCl, 1.0% IGEPAL CA-630, 0.5% sodium deoxycholate, 0.1% SDS, 50 mM Tris, pH 8.0) containing Halt Protease and Phosphatase Inhibitors (Pierce) and proteins were solubilized at 4 °C for 1 h. For whole-cell blots cortical lysates were centrifuged in order to pellet debris and total protein in the lysate supernatant was quantified using the Micro BCA Assay Kit (Pierce). In all, 30 μg of total protein was subjected to SDS-PAGE then transferred to an Amersham Protran Nitrocellulose membrane (GE Healthcare). Membranes were blocked with 5% BSA in 1× Tris-buffered saline (TBS at pH = 7.4) for 1 h at room temperature and incubated overnight with primary antibodies in TBS with 0.05% Tween (TBS-T) at 4 °C. For detection of primary antibodies (including anti-GAPDH [Abcam ab9484], anti-Cx43 [Sigma C6219], and anti-Cx30 [Invitrogen 71-2200]), membranes were incubated with LiCOR IRDye secondary antibodies in TBS-T for 1 h at room temperature. Blots were developed using a LiCOR Odyssey CLx Imaging System. Band intensity was quantified using LiCOR Image Studio software and normalized to appropriate controls.

Membrane fractionation was modified from a protocol previously described^71^. Dissected cortical tissue was collected and frozen on dry ice. Tissue was weighed then homogenized in TBS (8x v/w; 0.05 M Tris, 0.138 M NaCl, 0.0027 M KCl, pH 7.4) with PhosStop (Sigma) followed by brief sonication. Samples were then ultracentrifuged (100,000×*g*; 20 min; Beckman TLA-100.2). Supernatant was collected as cytosolic fraction. Pellet was resuspended in RIPA buffer (0.05 M Tris-HCl, 0.15 M NaCl, 0.5% Sodium deoxycholate, 0.5% sodium dodecyl sulfate, 1% Triton X-100, pH 7.4) and homogenized using brief sonication. Samples were then ultracentrifuged (100,000×*g*; 20 min; Beckman TLA-100.2) and supernatant was collected as membrane fraction. Protein concentration across samples was equated using BCA assay (Fisher Scientific). Western blot was performed as described above for whole-cell lysates.

### RNAseq determination of cortical astrocyte transcriptomes

Adult *Aldh1l1*-cre/ERT2 x Ribotag mice (4 males and 4 females) were used to purify astrocyte RNA as previously described^59^. Tamoxifen (Sigma; 20 mg/ml) was administered i.p. for 5 consecutive days at 75 mg/kg. Experiments were performed 2 weeks after the final tamoxifen injection. RNA was collected from somatosensory cortex of naïve and stress (20-min swim, 90-min recovery in home cage) from *Aldh1l1*-cre/ERT2 × Ribotag mice. RNA purification was performed as previously described (PMID:28712653). Briefly, freshly dissected tissue was collected from each animal and individually homogenized. RNA was extracted from 20% of cleared lysate as input, with the remaining lysate incubated with anti-HA antibody (1:250; Biolegend #901514) with rocking for 4 h at 4 °C followed by the addition of magnetic beads (Pierce #88803) rocked overnight at 4 °C. The beads were washed three times in high salt solution. RNA was purified from the IP and the corresponding input samples (Qiagen Rneasy Plus Micro). RNA concentration and quality were assessed with nanodrop and Agilent 2100 Bioanalyzer. RNA samples with RNA Integrity Number (RIN) greater than eight were used for multiplexed library prep with RiboZero Gold (Epicentre, WI) to remove ribosomal RNAs and Illumina TruSeq RNA sample prep kit. Amplified double-stranded cDNA was fragmented into 125 bp (Covaris-S2, Woburn, MA) DNA fragments, which were (200 ng) end-repaired to generate blunt ends with 5′- phosphates and 3′- hydroxyls and adapters ligated. The purified cDNA library products were evaluated using the Agilent Bioanalyzer (Santa Rosa, CA) and diluted to 10 nM for cluster generation in situ on the HiSeq paired-end flow cell using the CBot automated cluster generation system. All samples were multiplexed into a single pool in order to avoid batch effects and sequenced using an Illumina HiSeq 2500 sequencer (Illumina, San Diego, CA) across 2 lanes of 69-bp-paired-end sequencing, corresponding to three samples per lane and yielding between 52 and 65 million reads per sample. Quality control was performed on base qualities and nucleotide composition of sequences. Alignment to the M. musculus (mm10) refSeq (refFlat) reference gene annotation was performed using the STAR spliced read aligner (23104886) with default parameters. Additional QC was performed after the alignment to examine: the level of mismatch rate, mapping rate to the whole genome, repeats, chromosomes, key transcriptomic regions (exons, introns, UTRs, genes), insert sizes, AT/GC dropout, transcript coverage and GC bias. Between 89 and 92% (average 90.4%) of the reads mapped uniquely to the mouse genome. We used the HTSeq program (https://htseq.readthedocs.io/en/master/) to derive total counts of read-fragments aligned to candidate gene regions with mouse mm10 (Dec 2011) refSeq as a reference and used as a basis for the quantification of gene expression. Only uniquely mapped reads were used for subsequent analyses. Differential expression analysis was conducted with R-project and the Bioconductor package limma-voom (24485249). Statistical significance of the differential expression was determined at false discovery rate (FDR) <0.05. Only genes with FPKM > 1 were considered for the analyses. RNAseq data submission to Gene Expression Omnibus (GEO) repository (GEO accession ID GSE126172; www.ncbi.nlm.nih.gov/geo).

### Lactate biosensors

Lactate and null biosensors were purchased from Sarissa Biomedical (Coventry, UK). In these experiments signal from the null sensor was subtracted from the Lactate sensor to ensure specificity of the acquired signal. Lactate biosensors (0.5 mm in length and 7 µm in diameter). Experimental recordings began after an equilibration period of up to 1 h. Lactate biosensors were calibrated at the start and end of each day of experimentation to known concentrations of l-lactate (10 µM, 100 µM, and 1 mM) to ensure no degradation occurred between the beginning and end of experiments.

### dnCX43 generation and characterization

The threonine-154-alanine mutation was introduced to CMV-msfGFP-Cx43 construct (monomeric superfolder Green Fluoresescent Protein appended via a short linker to the N-terminus of rat Cx43, Addgene plasmid Plasmid #69024). The resulting msfGFP-Cx43T154A coding sequence was then subcloned into the AAV-gfaABC plasmid resulting in the AAV-gfaABC-msfGFP-Cx43T154A plasmid (referred to as dnCx43 for brevity). Expression and effectiveness of CMV-msfGFP-Cx43 and CMV-msfGFP-Cx43T154A in blocking endogenous Cx43 mediated coupling was performed using confocal microscopy and dual whole-cell patch clamp in transfected mouse astrocytes.

### Tissue dissociation, astrocyte sorting, and qPCR

Briefly, mice expressing eGFP under the astrocyte-specific *Aldh1l1* promotor were used to sort astrocytes by fluorescence-activated cell sorting (FACS). Following decapitation, hippocampi from four mice were dissected and digested together for 90 min at 36 **°**C in a 35 mm petri dish with 2.5 ml of papain solution (1x EBSS, 0.46% d-Glucose, 26 mm NaHCO_3_, 50 mm EDTA and 75 U/ml DNase1, 300 units of papain, and 2 mm l-cysteine) while bubbling with 95% O2 and 5% CO_2_. Following digestion, the tissue was washed 4 times with ovomucoid solution (1x EBSS, 0.46% d-Glucose, 26 mm NaHCO_3_, 1 mg/mg ovomucoid, 1 mg/ml BSA, and 60 U/ml DNase 1). The tissue was then mechanically dissociated with fire-polished borosilicate pipettes of two different sizes. A concentrated ovomucoid solution was added to the cell suspension (1x EBSS, 0.46% d-Glucose, 26 mm NaHCO_3_, 1 mg/mg ovomucoid, 1 mg/ml BSA, and 60 U/ml DNase 1).

Following RNA amplification, primers were designed and tested in a standard PCR. Serial dilutions of DNA from the whole hippocampus were used to make a standard curve for each set of primers to ensure amplification factors near 2.0 in all primer pairs to ensure similar efficacy.

The primer pairs used for quantification are as follows:

**Table:** 

**Protein**	**Gene**	**Direction**	**Sequence**
Cx43	*GJA1*	Forward	GGCGTGAGGGAAGTACCCAA
		Reverse	GTGGAGTAGGCTTGGACCTTG
Cx30	*GJB6*	Forward	TGGGTGTTTTGCTTGAGTCT
		Reverse	CCGTGATCCACACCTTCCC
Cx26	*GJB2*	Forward	CGACCCATTTCGGACCAACC
		Reverse	GGAGTGTGCCCCAATCCATC
Cx36	*GJD2*	Forward	GGGGAATGGACCATCTTGGA
		Reverse	ACACCGTCTCCCCTACAATG
Arbp	*ARBP*	Forward	TCCAGGCTTTGGGCATCA
		Reverse	AGTCTTTATCAGCTGCACATCAC

Following validation with standard PCR, qPCR reactions were set up in triplicate, and included, per reaction, 1 μL of water, 2 μL of each primer stock (10 μM), and 10 μL of the master mix (Fast Start essential, DNA Green Master, Roche Diagnostics, Indianapolis Indiana), along with 1 ng (Cx43, Arbp) or 10 ng (Cx26, Cx36, Cx30, Arbp) DNA diluted in water to 5 μL. qPCR was run on negative and positive fractions from three groups of mice in a LightCycler 96 (Roche), with a thermocycle steps as follows: preincubation 5 min at 95 °C, 3 step amplification 10 s at 95 °C, 10 s at 55 °C, 30 s at 72 °C times 45, melting cycle ramp from 65 °C to 97 °C. Fluorescence was recorded in the middle of each elongation cycle and continuously during the melting cycle. Analysis was carried out using the ΔΔCT method to determine levels of RNA relative to housekeeping genes.

### Molecular biology and adeno-associated virus generation

The EGFP-C1 plasmid from Clontech was modified to include msfGFP tethered to either the N-terminus or the C-terminus of Cx43 via a short linker to make a GFP-tagged dominant-negative Cx43 and a functional Cx43, respectively. An additional dominant-negative point mutation was added to convert the 154th amino acid of the Cx43 sequence from threonine to alanine in the N-terminus GFP-tagged Cx43 using Quick Change mutagenesis and with the following primers: Forward 5′-GGC GGC TTG CTG AGA GCC TAC ATC ATC AGC ATC-3′; Reverse 5′-AGG ATG CTG ATG ATG TAG GCT CTC AGC AAG CCG CC-3′. This construct is subsequently referred to as dnCx43 and was verified by DNA sequencing.

Following HEK293 and hTert astrocyte culture experiments, the minimal GfaABC_1_D promoter driving dnCx43 was cloned into pZac2.1 for virus generation via the In-Fusion technique (Clontech). First, the sequence coding for dnCx43 was linearized out of the EGFP-C1 plasmid using the following primers: Forward 5′-ATAGGCTAGCCTCGAGG-CCACCATGGTGAGCAAGG-3′; Reverse 5′-CCGGGTCGACTCTAGATTAAATCTCCA-GGTCATCAGGC-3′. The PCR product was ligated into the pZac2.1 vector containing the minimal GfaABC_1_D promoter. The resulting vector was sent to the Penn Vector Core Gene Therapy Program (University of Pennsylvania) and was made into an adeno-associated virus of serotype 2/5 (AAV 2/5), with a titer of 4.36e12.

### HEK-293 cell culture experiments

HEK-293 cells were maintained in DMEM/F12 media with Glutamax (Invitrogen) supplemented with 10% fetal bovine serum and penicillin/streptomycin. Cells were incubated at 37 **°**C with humidified 95% air/5%CO_2_. Cells were split 1:10 every 2–3 days, when confluence reached above 70%. In all, 1–2 days before transfections, during regular cell splitting, 50–150 μl of cells were added to six-well plates. When plated cells were at 50–60% confluence, they were transfected with 200 ng plasmid cDNA, and the Qiagen Effectene tranfection kit; (2.4 μl enhancer, 3 μl effectene, and 37 μl EC buffer). The following morning, cells were split into 24-well plates with polylysine coated coverslips, incubated for 3–4 h for adhesion, and used for experiments.

For HEK-293 experiments, coverslips were placed on an Olympus IX71 inverted microscope in a buffer containing (in mM) 150 NaCl, 1 CaCl_2_, 1 MgCl_2_, 10 glucose, 10 HEPES with pH adjusted to 7.4. Cells with physically attached neighbors were patched with a borosilicate glass pipette filled with (in mM) 150 NaCl, 10 HEPES, 10 EGTA, 20 Alexa Fluor 546. Alexa dye was allowed to diffuse into the patched cell for 5 minutes and only cells with <20 MΩ access resistance were kept. At the end of 5 minutes, fluorescence was imaged with an IXON DV887DCS EMCCD camera (Andor Technology) and an epifluorescence condenser and Polychrome V monochromator (Till Photonics). Images were analyzed with ImageJ by drawing ROIs around both the patched and the paired cell bodies, measuring average fluorescence, subtracting background fluorescence, and calculating a ratio of florescence in the paired cell to that of the patched cell.

Additionally, to measure trafficking of constructs to the junctional membranes (i.e. those opposed to neighboring cells), we imaged HEK cells on a confocal microscope (Olympus, Fluoview 1000). A single plane was imaged with the 488 nm wavelength laser. To analyze this data, a line ROI was drawn through both the junctional membrane and an unopposed membrane (ImageJ) and the fluorescence along that line was measured using the plot profile function. A ratio of junctional fluorescence was calculated by dividing the peak fluorescence at the junctional membrane by the peak fluorescence at the non-junctional membrane (Supplementary Fig. 5c, d, g, h).

### hTert astrocyte culture and experiments

Astrocytes were cultured from 19 days old embryos (E19). Primary astrocytes from wild type C57Bl/6 mice were immortalized using human telomerase reverse transcriptase (hTERT) overexpression, as previously described (Thi et al., 2010). Briefly, hTERT cDNA was PCR amplified from its original construct hTERT-pGRN145 (ATCC, MBA-141; Manassas, VA) and subcloned into pLentiV5-EF1α vector (modified from original vector from Invitrogen, cat# V49610). Confluent cultures of WT and Cx43-null astrocytes were transduced with lentiviral particles containing hTERT cDNA. After overnight incubation, the mixture was replaced with Astrocyte medium (ScienCell, cat#1801). Selection of hTERT-expressing cells was then achieved by successive splitting bi-monthly for passage 5 in culture, thus eradicating cells that did not continue to divide.

To overexpress the functional or dominant-negative connexin constructs, the hTERT-immortalized astrocytes were transfected using Optifect (Thermo Fisher, cat# 12579-017) according to the manufacturer’s instructions. Each 3.5 cm dish with 50–80% confluent astrocyte cultures were transfected with 4 µg of plasmid pre-mixed with 12 µl of optifect in Optimem (Thermo Fisher, cat# 31985070) and applied to the cells for 3 h. The media was changed back to Astrocyte media 3 hours after transfection. Astrocytes were later plated onto 12 mm round glass coverslips and after they adhered to the coverslip (2–3 h), they were used for patch clamp experiments. Cells were used 24–48 hours after transfection.

hTert astrocyte pairs were used for dual patch-clamp experiments, performed at room temperature in an extracellular buffer containing (in mM) 130 NaCl, 1 KCl, 3 CaCl_2_, 5 HEPES, and 10 d-glucose. Neighboring astrocytes with at least one expressing the fluorescent construct were patched with borosilicate pipettes (World Precision Instruments) pulled to a resistance of 5–7 MΩ and filled with (in mM) 130 CsCl, 0.01 EGTA, 0.01 HEPES, 5 CaCl_2_, and 5 Na-ATP. Signals were detected with an Axopatch 1D amplifier (Molecular Devices), digitized by and Axon Instruments digitizer and acquired with Clampex 6.0 software (Molecular Devices). Once two physically attached astrocytes were patched with <20 MΩ access resistance in each pipette, inter-cellular conductance was measured with a voltage ramp protocol from +100 to −100 mV applied to only one of the cells, while the second cell was held at −60 mV. Current in both cells was recorded, and junctional conductance in the second cell was calculated offline as current response in the second cell as a ratio of voltage changes in the first cell (Clampex, Molecular Devices).

### Surgery and in vivo microinjections of AAV 2/5

Mice (C57bl/6, P42-P50), were anesthetized via isoflurane (5% for induction, 2–3% for maintenance, v/v). Depth of anesthesia was determined by observing breathing rates and toe-pinch ensured proper loss of reflexes. Following deep anesthesia, mice were head fixed on a stereotaxic apparatus (David Kopf Instruments) with a bite bar and ear bars, with ventilated anesthesia administration. In all, 0.05 μl of buprenorphine was injected subcutaneously (Buprenex, 0.1 mg/mL), and artificial tears were applied to the eyes before beginning surgery. The hair on the scalp was removed prior to surgery, and the incision was washed with 10% povidone iodine and 70% ethanol, three times each, alternating. An incision was made on the scalp to expose bregma and the craniotomy site with coordinates are as follows. Ventral hippocampus: 2.9 mm posterior and 2.9 mm lateral from bregma, and 3.1 mm ventrally from the pial surface; Dorsal hippocampus: 2.1 mm posterior and 2.0 mm lateral from bregma, and 1.8 mm ventrally from the pial surface. A 2–3-mm craniotomy was made at the injection site using a small burr (Fine Science Tools), powered by a drill (K.1070, Foredom). Saline (0.9%) was applied to keep the skull cool, to maintain skin hydration, and to remove bone debris. AAVs were injected via a beveled borosilicate pipette (World Precision Instuments) at an infusion rate of 200 nL/min by a mechanical pump (Pump11 Pico-Plus Elite, Harvard Apparatus). In all, 1 μl of virus was infused into the left hippocampus, and each virus contained the minimal GfaABC_1_D promoter driving the following constructs at the indicated titer: dnCx43 (as described above, 4.36e12 gC/mL), tdTomato (tdT, Penn vector core, 4.96e13 gC/mL); cytosolic-GFP, (1e13 gC/mL), lck-GFP (Shigetomi et al. 2013, 2.41e13 gC/mL). Following injection, the needle was left in place for 10 min to allow for fluid pressure normalization. Following needle withdraw, scalp was sutured with silk sutures and mice were closely monitored, kept on a heating pad and given buprenorphine twice daily for 48 h post-op (0.05 mL, 0.1 mg/mL), and fed chow with sulfonamide sulfadiazine trimethoprim (32 g/Kg) for 1 week post-op. Experiments were performed 2–4 weeks post-injection.

### Immunohistochemistry

To prepare fixed brain sections, mice were euthanized by an intraperitoneal overdose of pentobarbital (0.6 mL of 10 mg/ml). Once the toe pinch reflex was abolished, mice were transcardially perfused with PBS for 5 min followed by 10% formalin for 10 min. The brain was then removed and placed in 10% formalin over night. The following day, brains were transferred to 30% sucrose solution in PBS for dehydration for 2–3 days. Brains were sectioned into 40-μ-thick sections on a cryostat (Leica) and processed for immunohistochemistry. Sections were first washed three times in PBS, then placed in a blocking solution of 10% normal goat serum and 0.5% Triton X-100 in PBS for 90 min. Sections were then placed in primary antibody in 0.5% Triton X-100 overnight at 4 °C. The following day, sections were again washed three times in PBS, then placed in secondary antibody in PBS for 2 hours. Sections were washed again three times, mounted on glass slides, dried, covered in Fluoromount (Southern Biotech) and coverslipped. Antibody pairs used are as follows: Chicken anti-GFP (1:1000, Abcam, ab13970) and goat anti-chicken Alexa 488 (1:1000, Invitrogen, A11039), Mouse anti-Cx43 (1:500, BD Biosciences, 610061) and goat anti-mouse 546 (1:1000, Invitrogen, A11003), Rabbit anti-NeuN (1:2000, Cell Signaling, D3S31) and goat anti-rabbit 546 (1:1000 Invitrogen, A11010), rabbit anti-S100β (1:1000, Abcam, ab41548) and goat anti-rabbit 546 (1:1000, Invitrogen, A11010). Sections were imaged on an Olympus Fluoview 1000 upright confocal microscope and analyzed in ImageJ.

### Dye-spread coupling assay

To measure coupling between astrocytes expressing either dnCx43 or cytosolic-GFP AAV, astrocytes were patched with K-gluconate intracellular solution with 2 mg/mL biocytin added. Biocytin was allowed to dialyze into the syncytium for 20 min before the pipette was removed and the slice was immediately rescued and placed in 10% formalin. Following overnight fixation, sections were permeabilized with 0.2% Triton-X in PBS with 10% Normal Goat Serum (NGS). These sections were then placed in Chicken anti-GFP primary antibody, (AbCam ab13970, 1:1000) overnight, shaking, at 4 **°**C. The following day sections were rinsed with PBS and placed in secondary antibody (Goat anti-chicken 488, Invitrogen, 1:1000) and streptavidin-conjugated Alexa-Fluor 555 (Invitrogen, 1:1000) for 2 hours. Sections were again rinsed and mounted in fluoromount (Southern Biotech). Sections were imaged on an Olympus Confocal microscope with a ×20 oil objective to image the entire spread of biocytin. Cell bodies positive for streptavidin-conjugated Alexa-Fluor 555 were counted to determine the extent of coupling.

### Astrocyte morphology

To examine the detailed morphology of CA3 astrocytes, cells were iontophoretically filled with fluorescent dye. Mice were given an overdose of pentobarbital and once all reflexes had subsided, the chest cavity was opened. The heart was injected first with 0.02 mL heparin to reduce blood clotting before perfused transcardially first with warm, oxygenated Ringer’s solution (in %w/v, 0.79 NaCl, 0.038 KCl, 0.02 MgCl2, 0.018 Na2HPO4, 0.125 NaHCO3, 0.03 CaCl2, 0.2 d-glucose, 0.02 lidocaine), followed by 10% buffered formalin for 5 min. The brain was removed and placed in post-fixative for 1.5 h, then sliced to sections of 110 μm on a vibrating microtome (Vibratome 3000). Astrocytes expressing either dnCx43 or lck-GFP AAV were visualized on an Olympus laser-scanning confocal microscope (FV1000). Cell bodies were visualized as ‘holes’ in the membrane bound fluorescence. Cell bodies were punctured with sharp, borosilicate micropipettes filled with KCl intracellular solution containing 10 μM Alexafluor 568. Dye was injected into the cell body with a stimulus isolator (A365, World Precision Instruments). Once all processes in the territory were visible (at lease 5 min), the electrode was removed and the cell was imaged at 0.5-μm z-steps (Fluoview, Olympus). Off-line, cell and territory volumes were quantified using Imaris software (Bitplane). Cell volumes were calculated by creating volumes from three compartments; soma, branches, and processes, and detecting background-subtracted fluorescence. The entire territory was analyzed separately. To determine soma size a surface was created in Imaris using a fluorescence threshold and minimum size detection. The soma was trimmed to exclude any major branches, and the soma surface was masked for branch detection. Major branches were similarly detected in the masked channel by again applying a fluorescence threshold, as well as a particle size threshold to minimize inclusion of small processes. This volume was again masked for process detection. Processes were detected in the double masked channel with a method similar to that for branch detection, but with a smaller volume threshold. Territory was analyzed by using the original fluorescence image, an absolute intensity threshold, and a large size for the volume calculation.

### Statistical analysis

Prior to statistical comparison, normality test as well as variance analysis were performed, and the appropriate two-sided statistical parametric or nonparametric test was used. When only two groups were generated a two-tailed Student’s t test was used for normally distributed data, a Mann–Whitney U-test was used to compare pairs of non-normally distributed datasets. For multiple groups a one-way ANOVA was used with appropriate post-hoc tests for comparison between groups. Appropriate sample sizes were based on best practices in the literature as well as on ethical standards to minimize numbers of animals for experiments and were dictated by the magnitude of experiment- to experiment variation. Statistical analysis was performed in GraphPad Prism (GraphPad Software, USA) and Ingenuity Pathway Analysis (Qiagen). There was no explicit blinding or randomization.

### Reporting summary

Further information on research design is available in the Nature Research Reporting Summary linked to this article.

## Supplementary information

Supplementary Information

Peer Review File

Description of Additional Supplementary Files

Supplementary Movie 1

Supplementary Movie 2

Supplementary Data 1

Supplementary Data 2

Reporting Summary

## Data Availability

RNAseq data is available at the Gene Expression Omnibus (GEO) repository (GEO accession ID GSE126172; www.ncbi.nlm.nih.gov/geo). Source data are provided are source data file. All other data that support the findings of this study are available from the corresponding author upon request.
