# Author Correction: Stress gates an astrocytic energy reservoir to impair synaptic plasticity

**DOI:** 10.1038/s41467-020-16668-w

**Published:** 2020-06-11

**Authors:** Ciaran Murphy-Royal, April D. Johnston, Andrew K. J. Boyce, Blanca Diaz-Castro, Adam Institoris, Govind Peringod, Oliver Zhang, Randy F. Stout, David C. Spray, Roger J. Thompson, Baljit S. Khakh, Jaideep S. Bains, Grant R. Gordon

**Affiliations:** 1 Hotchkiss Brain Institute, Cumming School of Medicine, Department of Physiology and Pharmacology, University of Calgary, Calgary, Canada; 2 Department of Physiology, David Geffen School of Medicine, University of California Los Angeles, UCLA, Los Angeles, CA USA; 3 Department of Biomedical Sciences, New York Institute of Technology College of Osteopathic Medicine, Old Westbury, NY USA; 4 Dominick P. Purpura Department of Neuroscience, Albert Einstein College of Medicine, Bronx, NY USA

**Keywords:** Astrocyte, Stress and resilience, Long-term potentiation

Correction to: *Nature Communications* 10.1038/s41467-020-15778-9, published online 24 April 2020.

The original version of the Supplementary Information associated with this Article contained an error in the Supplementary Data 2 file, which arose during manuscript preparation. The values in column D were sorted in descending order, but columns A–C were not re-ordered accordingly. The HTML has been updated to include the correct Supplementary Data 2 file.

