# Supplementary material for: Stress gates an astrocytic energy reservoir to impair synaptic plasticity
Source: Nat Commun. 2020 Apr 24;11:2014. doi: 10.1038/s41467-020-15778-9 (PMC7181611; doi:10.1038/s41467-020-15778-9)
Supplement: Supplementary file 1 — Supplementary Information [file 41467_2020_15778_MOESM1_ESM.pdf]

Supplementary information – Murphy-Royal et al. "Stress gates an astrocytic energy reservoir to impair synaptic plasticity".

Supplementary Figure 1

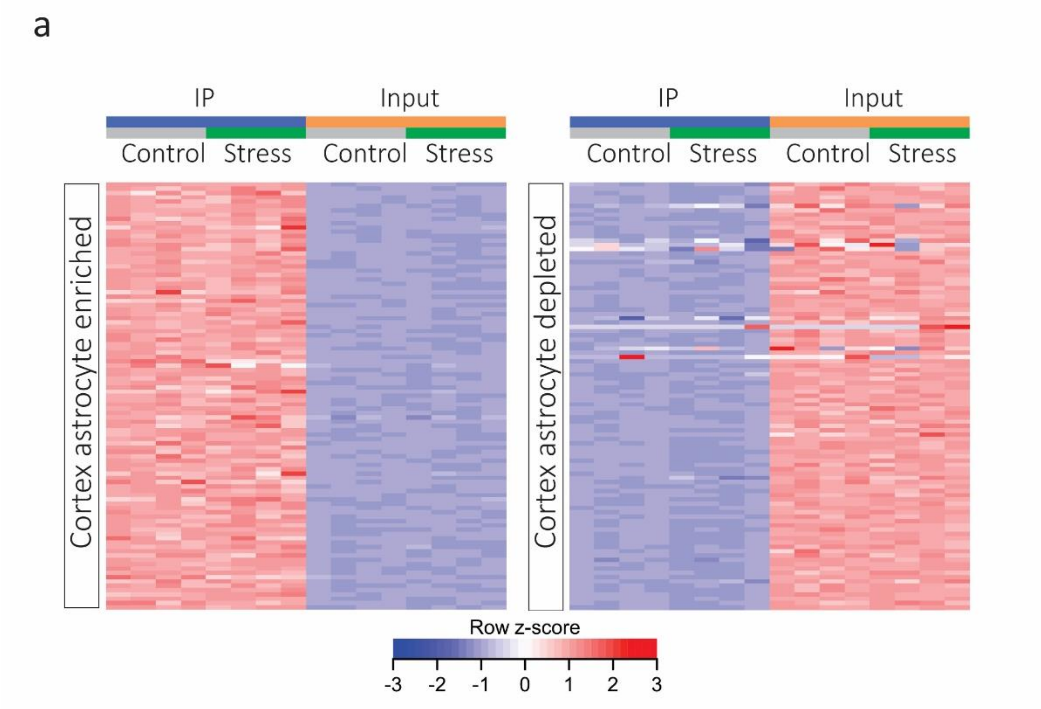

(a) Heat map, depicting changes in astrocyte enriched and astrocyte depleted genes in total RNA (INPUT) as well as specifically in astrocytes (IP) in naïve (control) and stress conditions.

Supplementary Figure 2

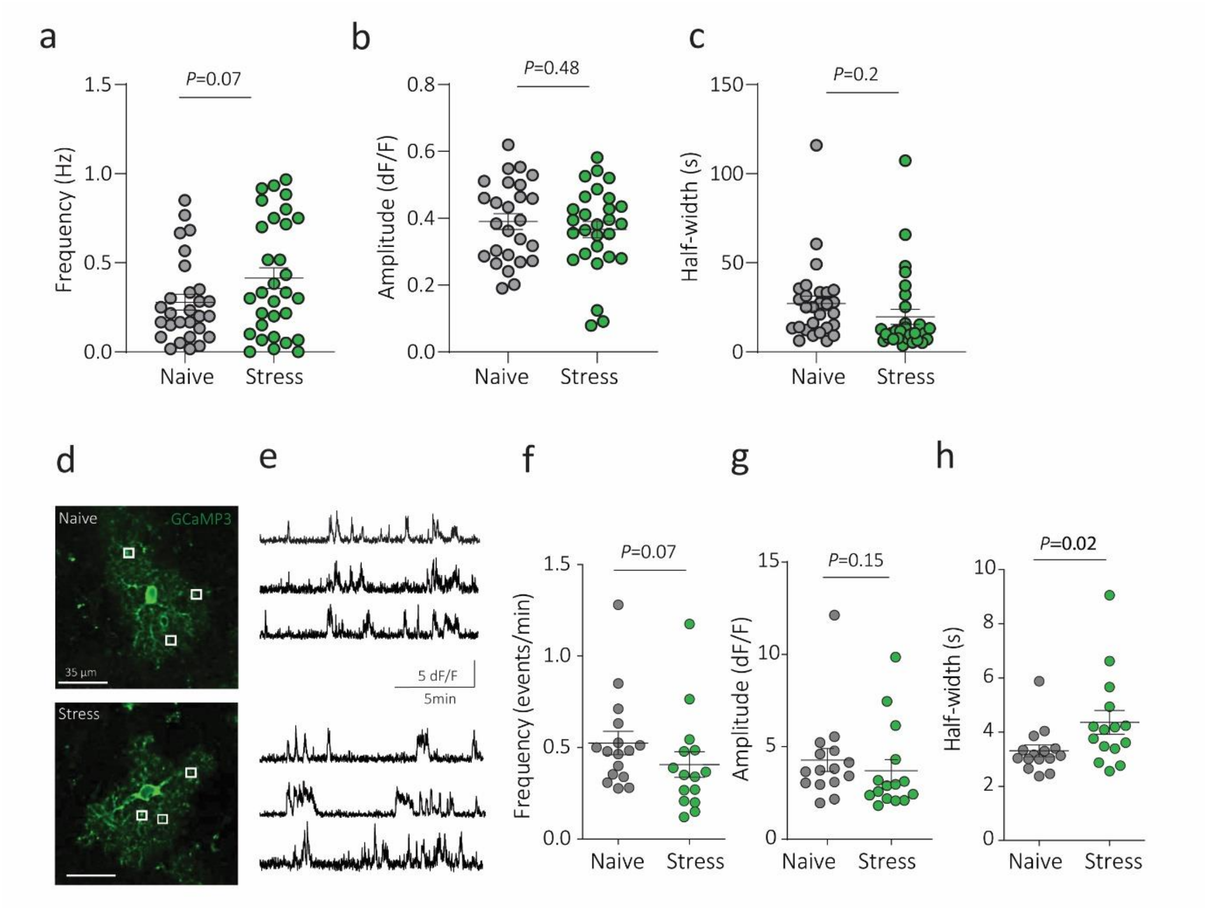

(a) No effect of stress on the frequency of calcium events in astrocyte soma in Aldh1l1-GCaMP6s mice. n=3 mice, n=24 cells. Error bars: mean±s.e.m. Unpaired t-test, *P*=0.07. **(b)** No effect of stress on the amplitude of calcium events in astrocyte soma in Aldh1l1-GCaMP6s mice. n=3 mice, n=24 cells. Error bars: mean±s.e.m. Unpaired t-test, *P*=0.48. **(c)** No effect of stress on the duration (half-width) of calcium events in astrocyte soma in Aldh1l1-GCaMP6s mice. n=3 mice, n=24 cells. Error bars: mean±s.e.m. Unpaired t-test, *P*=0.2. **(d)** Representative images of GCaMP3-expressing astrocytes in naïve and stress conditions. Scale bar 35µm. **(e)** Representative raw traces of calcium activity in naïve (top) and stress (bottom) Scale bars 5df/f and 5min. **(f)** Scatter dot plot comparing frequency of calcium events between naïve (n=3 mice, n=15 cells) and stress (n=3 mice, n=15 cells) conditions. Error bars: mean±s.e.m. Mann-Whitney U. **(g)** Scatter dot plot comparing the effects of stress on the amplitude of individual calcium events. Error bars: mean±s.e.m. Mann-Whitney U. **(h)** Scatter dot plot comparing half-width of individual calcium events between naïve and stress. Error bars: mean±s.e.m. Mann-Whitney U.

Supplementary Figure 3

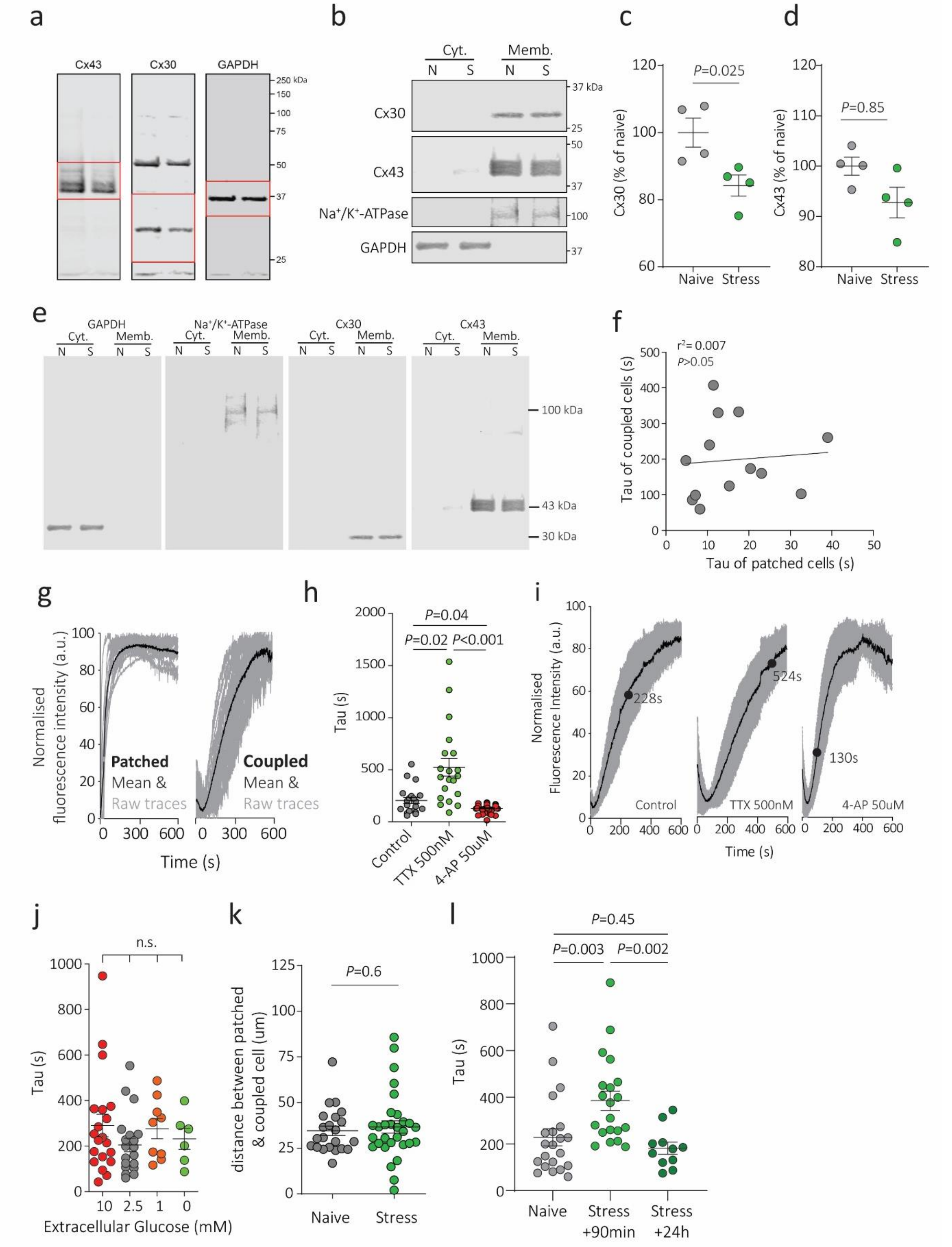

(a) Full-length blots. Red box indicates cropped data represented in Fig. 3 a-c. **(b)** Representative blots depicting enrichment of membrane fractions following a membrane

enrichment protocol from cortices of naïve and stressed mice. Anti-Na/K-ATPase antibody was used to validate membrane enrichment. **(c)** Protein expression of Cx30 in the membrane fraction was significantly reduced after stress. n=4 mice per group. Error bars: mean±s.e.m. Unpaired t-test. **(d)** Protein expression of Cx43 in the membrane fraction was not changed after stress. n=4 mice per group. Error bars: mean±s.e.m. Unpaired t-test. **(e)** Full-length blots of membrane fractionation data. **(f)** No correlation exists between tau of patched and coupled cells (n=7 mice, n=13 pairs of cells). Pearson's correlation coefficient. **(g)** Raw (grey) and average (black) traces of dye filling in patched (left) and coupled (right) astrocytes. **(h)** Astrocyte coupling bi-directionally responds to neuronal activity (Control tau=228.6±36.9s, n=6 mice, n=21 cells; TTX tau=524.6±84.7s, n=3 mice, n=20 cells; 4-AP tau=130.4±5.7s, n=9 mice, n=42 cells; mean±s.e.m.). Error bars: mean±s.e.m. One-way ANOVA. **(i)** Mean trace of coupling in each group with tau value indicated. Error indicates standard deviation. **(j)** No effect of acute modifications in extracellular glucose concentration. 10mM n=20cells tau=290.1±49.5s; 2.5mM n=21 cells, tau=228.6±36.9s; 1mM n=9 cells, tau=276.5±44.2s; 0mM n=6 cells, tau=231.8±46.1s, mean±sem; Error bars: mean±s.e.m. one-way anova with Tukey's multiple comparison test. $F(3,52)=0.467$, $P=0.71$. **(k)** No effect of stress on the mean distance between patched and coupled cells. Naïve - n=22 pairs of cells, mean distance=34.5±2.6µm; Stress - n=30 pairs of cells, mean distance=36.6±3.4µm, mean±s.e.m. Error bars: mean±s.e.m. Mann-Whitney test, U=300.5, df=50, $P=0.6$. **(l)** The effects of acute stress on astrocyte coupling is transient. One-way ANOVA F=7.19 P=0.0018. Naïve: n=21cells tau=228.6±36.9s; Stress+90min: n=20 cells, tau=385.1±42s; Stress+24h: n=11cells, tau=182.2±26s, mean±s.e.m. Error bars: mean±s.e.m.

Supplementary Figure 4

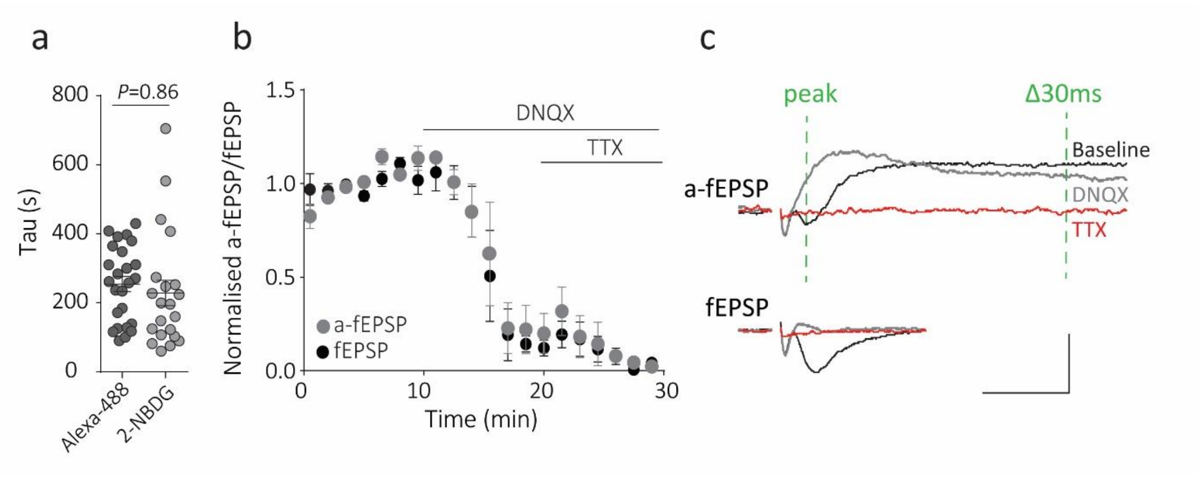

(a) No difference in dye coupling between astrocytes when using either Alexa-488 (tau=254.5±21.9s; n=7 mice, n=25 cells) or 2-NBDG (tau=228.6±36.9s; n=6 mice, n=21 cells; Mann-Whitney U=242, *P*=0.86). Error bars: mean±s.e.m. **(b)** Normalized fEPSP and a-fEPSP demonstrating the sensitivity to DNQX (50μM) and TTX (1μM; n=2 mice, n=3 cells). Error bars: mean±s.e.m. **(c)** Example traces of a-fEPSP and fEPSPs in baseline, DNQX, and TTX. Scale bars:0.5mV,10ms.

Supplementary Figure 5

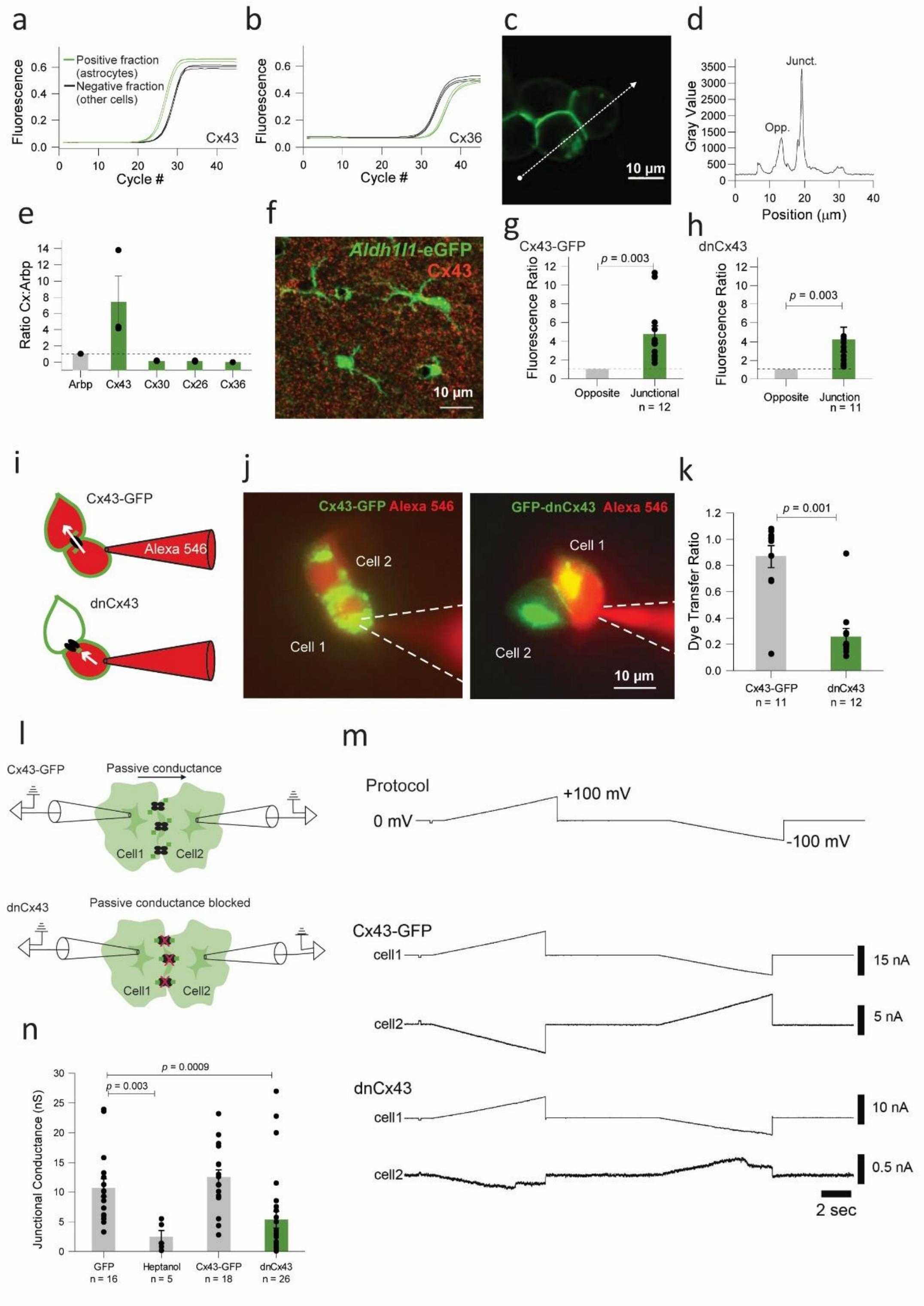

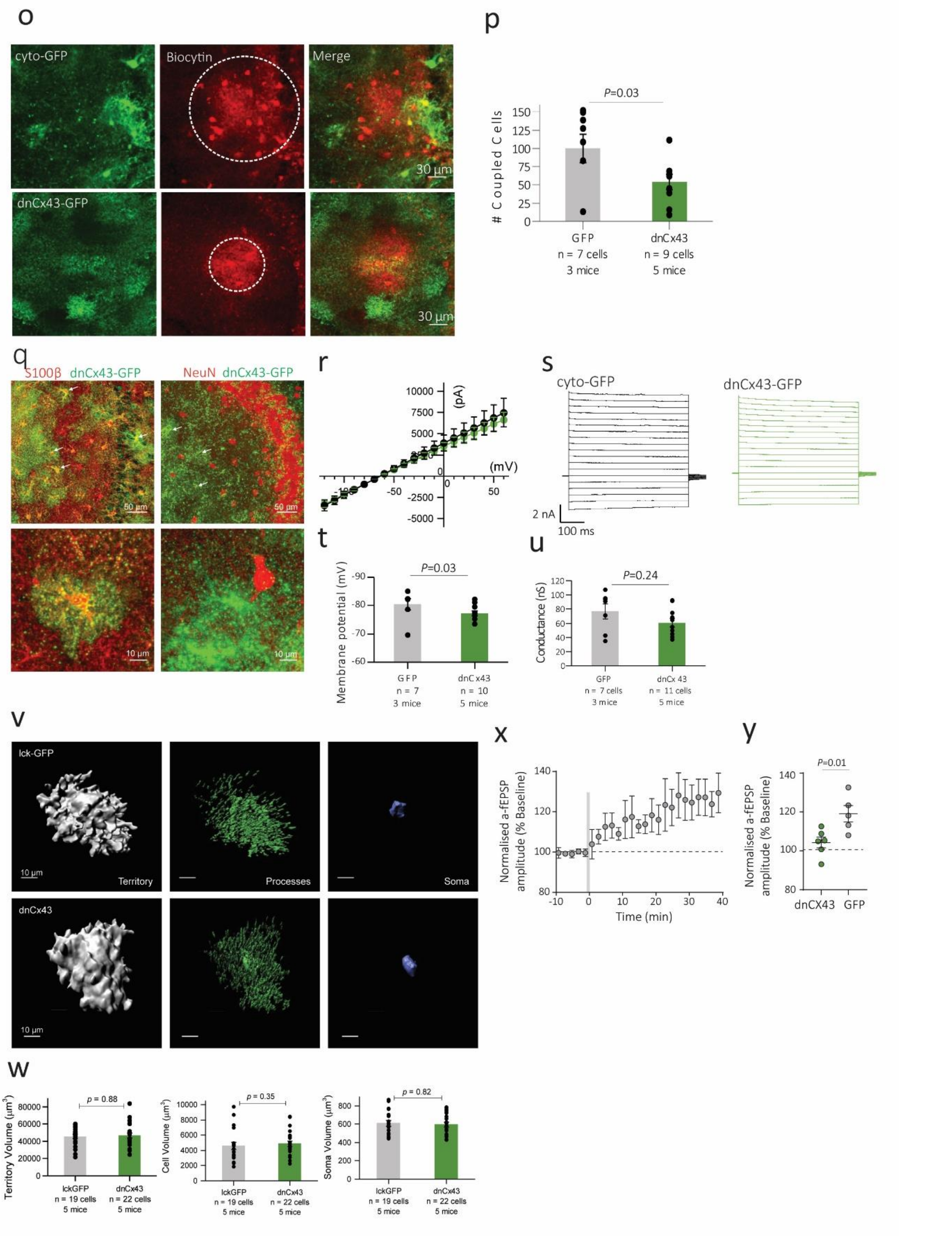

(a) Enrichment of Cx43 in astrocyte fractions compared to other cell types as assessed by qPCR. **(b)** Lack of enrichment of Cx36, a neuronal connexin, in astrocyte fractions compared to other cell types as assessed by qPCR. **(c)** dnCx43, expressed in HEK293 cells, traffics to junctional membranes. **(d)** Representative image depicting increased trafficking of dnCx43 to junctional membranes as assessed by fluorescence due to GFP expression. **(e)** Enrichment ratio of Cx43, Cx30, Cx26, and Cx36 to the housekeeping gene Arbp in astrocyte fractions following qPCR.

Error bars: mean±s.e.m. **(f)** Immunohistochemical images of Cx43 antibodies in Aldh1l1-GFP mice. **(g)** Quantification of the ratio of junctional to opposite membrane fluorescence in HEK293 cells expressing a non-dnCx43-GFP. Unpaired t-test. Error bars: mean±s.e.m. **(h)** Quantification of the ratio of junctional to opposite membrane fluorescence in HEK293 cells expressing dnCx43. Error bars: mean±s.e.m. Unpaired t-test. **(i)** Schematic representation of dye coupling experiments in cells expressing Cx43-GFP and dnCX43**. (j)** Representative images depicting impairment of dye flux between cells expressing dnCX43 compared to control (Cx43-GFP). **(k)** Quantification of dye coupling in cells expressing Cx43-GFP and dnCX43. Mann-Whitney U test. Error bars: mean±s.e.m. **(l)** Schematic representation of electrical conductance experiments carried out by patching two coupled astrocytes and measuring conductance mediated by gap-junction channels **(m)** *Top* – protocol of electrical conductance experiment. *Middle* – Representative traces demonstrating robust passive electrical conductance between astrocytes in cells expressing control virus (Cx43-GFP). *Bottom* – Representative traces demonstrating impaired passive electrical conductance between astrocytes expressing dnCX43. **(n)** Quantification of passive electrical coupling between astrocytes in control (GFP expressing astrocytes), in the presence of gap-junction channel blocker heptanol, in Cx43-GFP expressing astrocytes, and astrocytes expressing dnCX43. One-way ANOVA. Error bars: mean±s.e.m. **(o)** Representative images of astrocyte syncytial filling experiments. *Top* – Extensive syncytial filling in astrocytes expressing cytosolic-GFP (control virus). *Bottom* – Size of astrocytic network is reduced when cells express dnCX43. **(p)** Quantification of astrocyte network size (number of coupled cells in control (GFP) and dnCX43 expressing astrocytes. Unpaired t-test. Error bars: mean±s.e.m. **(q)** Immunostaining showing colocalization of CX43-GFP with astrocyte specific marker S100B (*left panels)* but no colocalization with neuronal marker NeuN (*right*). **(r)** Average current-voltage relationships (IV curves) in astrocytes expressing dnCx43 (green), or a control virus (GFP, black). **(s)** Representative traces of responses to voltage steps in astrocytes expressing either dnCx43 (green), or a control virus (GFP, black). **(t)** Astrocytes expressing dnCx43 (green) rest at a slightly depolarized resting membrane potential as compared to those expressing the control virus (GFP, gray). Unpaired t-test. Error bars: mean±s.e.m. **(u)** Astrocytes expressing dnCx43 (green) display no change in conductance when compared to those expressing the control virus (GFP, gray). Unpaired t-test. Error bars: mean±s.e.m. **(v)** Single astrocyte morphology assessed in control (lck-GFP) and dominant-negative Cx43-expressing astrocytes. Scale bar 10um. **(w)** Expression of dnCX43 does not affect astrocyte morphology. Unpaired t-tests. Error bars: mean±s.e.m. **(x)** Normalized a-fEPSP amplitude of LTP recorded through astrocytes virally expressing eGFP. Error bars: mean±s.e.m. **(y)** Comparison of LTP in dnCX43 and GFP (control) expressing astrocytes. Demonstrating minimal impact of GFP expression on synaptic plasticity. Unpaired t-test, *P*=0.01. Error bars: mean±s.e.m.

Supplementary Figure 6

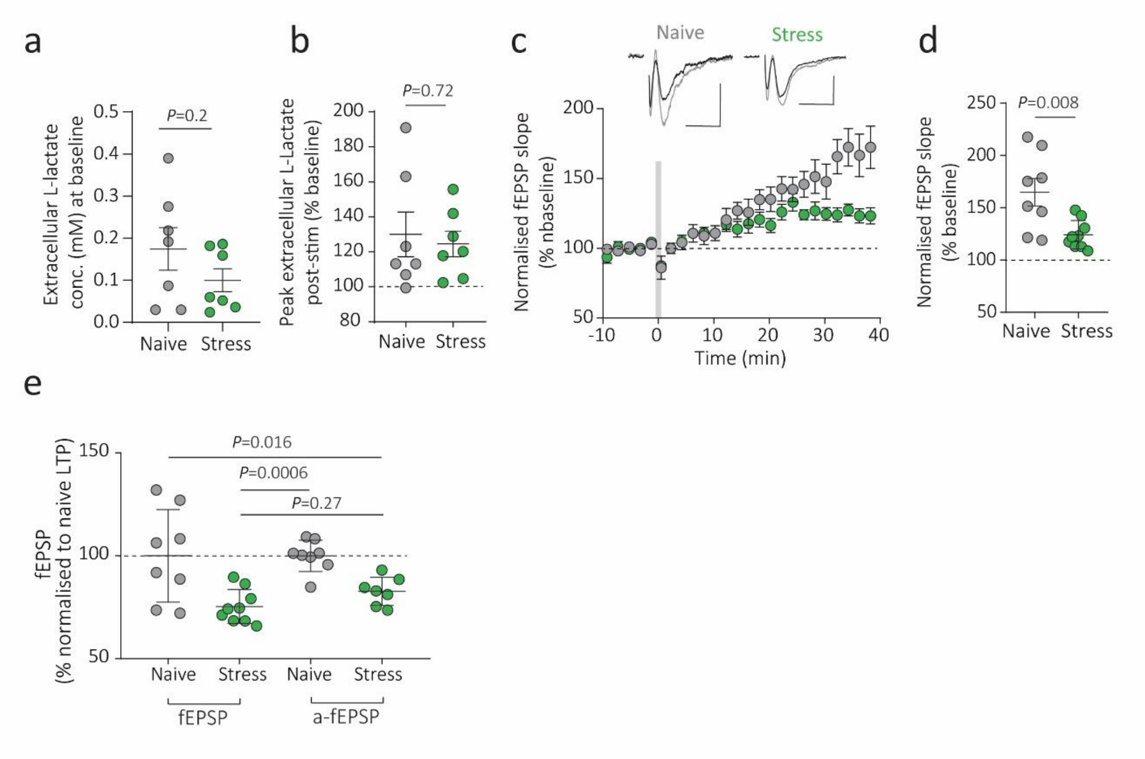

(a) No difference in basal extracellular L-lactate concentration was observed using enzymatic probes. Naïve: n=3 mice, n=7 brain slices, L-lac=0.18±0.05mM; Stress: n=3mice, n=7 brain slices, L-lac=0.099±0.03; Unpaired t-test, t=1.3, df=12, *P*=0.22. Error bars: mean±s.e.m. **(b)** Scatter dot plot depicting peak extracellular L-lactate immediately after theta burst stim in naïve and stress conditions. Naïve - 130±12.8%, Stress - 124.5±7.3%; Unpaired t-test, t=0.37, df=12, *P*=0.72. Error bars: mean±s.e.m. **(c)** fEPSP recordings from neocortex demonstrating impaired LTP following a single bout of acute swim stress. Grey vertical bar denotes 10s theta burst stim. Error bars: mean±s.e.m. *Inset* average traces of fEPSP at baseline and 30-40min following theta burst stim. Scale bars depict 0.25mV and 10ms. **(d)** Comparison of LTP between naïve and stress group. Each point is the average of the 30-40min time period from each recording. Error bars: mean±s.e.m. **(e)** Comparison of LTP (normalized to Naïve LTP) recorded by classical extracellular fEPSP compared with intracellular a-fEPSP. fEPSP – naïve=100±7.9%, stress=75.3±2.7%; a-fEPSP – naïve=100±2.7%, stress=82.8±2.6% mean±s.e.m. Error bars: mean±s.e.m.

Supplementary Methods

Preparation of brain slices for electrophysiology. Briefly, mice were anesthetized with inhalant isoflurane, quickly decapitated and the brain was removed and the sectioned at 300-350 µm (DSK Zero-1 microslicer) in ice-cold sucrose slicing buffer containing (in mM) 194 sucrose, 30 NaCl, 4.5 KCl, 1.2 NaH_2PO_4, 26 $NaHCO_3$, 10 D-glucose, 1 $MgCl_2$. Sections were maintained in an interface style holding chamber containing aCSF (in mM; 124 NaCl, 4.5 KCl, 1.2 NaH_2PO_4, 26 $NaHCO_3$, 10 D-glucose, 2 $CaCl_2$, 1 $MgCl_2$, saturated with 95% O_2, 5% CO_2) at ~33°C for 30 min following slicing and then at room temperature for patch-clamp experiments, and at ~33°C continually for LFP recordings. To confirm virus expression, sections were postfixed in 10% formalin for 24 hours and then either mounted immediately (tdT) or immunolabeled for GFP following the protocol listed above (dnCx43, cyto-GFP, lck-GFP) before mounting. Sections were imaged with 5X magnification on an upright epifluorescence microscope (Zeiss).

Whole cell electrophysiological recordings. Whole cell patch-clamp recordings were made from astrocytes using borosilicate pipettes pulled to a resistance of 4-6 MΩ and filled either with K-gluconate intracellular solution (in mM; 135 K-gluconate, 3 KCl, 10 HEPES, 1 EGTA, 0.1 $CaCl_2$, 4 Mg-ATP, 0.3 Na-ATP, 8 Na_2-phosphocreatine), KCl intracellular (in mM; 138 KCl, 10 HEPES, 1 EGTA, 0.1 $CaCl_2$, 4 Mg-ATP, 0.3 Na-ATP, 8 Na_2-phosphocreatine), or Cs-methansulfonate intracellular (in mM; 120 $CsMeSO_3$, 15 CsCl, 8 NaCl, 10 HEPES, 0.2 EGTA, 0.1 $CaCl_2$, 2 Mg-ATP, 0.3 Na-ATP, 10 TEA-Cl). For all whole-cell recordings, only cells with access resistance below 20 MΩ were kept. Cells were visualized using DIC on an Olympus BX50WI microscope with a 40X objective. Signals were recorded using a Multiclamp amplifier (700A, Molecular Devices) and a Digidata digitizer (1322A, Molecular Devices), with Clampex 9 software. Current clamp experiments were recorded at resting membrane voltage, with the exception of a ramp from -180 to 60 pA to determine rheobase.
