# Supplementary material for: Stress gates an astrocytic energy reservoir to impair synaptic plasticity
Source: Nat Commun. 2020 Apr 24;11:2014. doi: 10.1038/s41467-020-15778-9 (PMC7181611; doi:10.1038/s41467-020-15778-9)
Supplement: Supplementary file 3 — Description of Additional Supplementary Files [file 41467_2020_15778_MOESM3_ESM.docx]

Description of Additional Supplementary Files

**Supplementary Movie 1:** Real-time astrocyte-astrocyte coupling experiment. Astrocytes were first identified using SR101 dye uptake, groups of at least two astrocytes in a single imaging plane were then selected. One of the astrocytes in the region of interest was patch-filled with a fluorescent dye (Alexa-488 or 2-NBDG) and dye flux to neighbouring astrocytes was recorded.

**Supplementary Movie 2:** Astrocyte coupling experiment in the presence of gap-junction channel blocker carbenoxalone. To note in this movie - dye flux from patched astrocyte to neighbouring astrocytes is completely absent when gap-junction channels are blocked.

**Supplementary Data 1:** RNAseq analysis comparing naïve to stressed astrocytes. IP denotes immunoprecipitate, i.e. RNA from Ribotag-expressing astrocytes. INPUT represents pooled RNA from brain tissue homogenate.

**Supplementary Data 2:** Table describing top features used by classifier to distinguish differences between naïve and stressed astrocyte microdomain calcium.
