# Supplementary material for: Stress gates an astrocytic energy reservoir to impair synaptic plasticity
Source: Nat Commun. 2020 Apr 24;11:2014. doi: 10.1038/s41467-020-15778-9 (PMC7181611; doi:10.1038/s41467-020-15778-9)
Supplement: Supplementary file 8 — Reporting Summary [file 41467_2020_15778_MOESM8_ESM.pdf]

nature research

Corresponding author(s): Jaideep S. Bains & Grant R. Gordon

Last updated by author(s): Mar 14, 2020

Reporting Summary

Nature Research wishes to improve the reproducibility of the work that we publish. This form provides structure for consistency and transparency in reporting. For further information on Nature Research policies, see Authors & Referees and the Editorial Policy Checklist .

Statistics

For all statistical analyses, confirm that the following items are present in the figure legend, table legend, main text, or Methods section.

n/a	Confirmed	
☐	☒	The exact sample size (n) for each experimental group/condition, given as a discrete number and unit of measurement
☐	☒	A statement on whether measurements were taken from distinct samples or whether the same sample was measured repeatedly
☐	☒	The statistical test(s) used AND whether they are one- or two-sided<br>*Only common tests should be described solely by name; describe more complex techniques in the Methods section.*
☐	☒	A description of all covariates tested
☐	☒	A description of any assumptions or corrections, such as tests of normality and adjustment for multiple comparisons
☐	☒	A full description of the statistical parameters including central tendency (e.g. means) or other basic estimates (e.g. regression coefficient) AND variation (e.g. standard deviation) or associated estimates of uncertainty (e.g. confidence intervals)
☐	☒	For null hypothesis testing, the test statistic (e.g. F, t, r) with confidence intervals, effect sizes, degrees of freedom and P value noted<br>*Give P values as exact values whenever suitable.*
☒	☐	For Bayesian analysis, information on the choice of priors and Markov chain Monte Carlo settings
☒	☐	For hierarchical and complex designs, identification of the appropriate level for tests and full reporting of outcomes
☒	☐	Estimates of effect sizes (e.g. Cohen's d, Pearson's r), indicating how they were calculated

Our web collection on statistics for biologists contains articles on many of the points above.

Software and code

Policy information about availability of computer code

Data collection	Electrophysiology - pClamp 10.2 (Molecular Devices)<br>2P imaging - scanimage 4.2 (vidrio technologies)<br>Western blot - LiCOR Odyssey CLx Imaging System & image studio software<br>RNAseq - R-project and the Bioconductor package limma-voom (24485249) for differential expression analysis. Ingenuity software for canonical pathway analysis (Qiagen)<br>Image J<br>Matlab 2019b<br>Clampex 6.0<br>Clampex 9.0<br>Clampfit 10.6<br>Corel Draw 17
Data analysis	Prior to statistical comparison, normality test as well as variance analysis were performed and the appropriate two-sided statistical parametric or nonparametric test was used. When only two groups were generated a two-tailed Student's t test was used for normally distributed data, a Mann-Whitney U-test was used to compare pairs of non-normally distributed datasets. For multiple groups a one-way ANOVA was used with appropriate post-hoc tests for comparison between groups. Appropriate sample sizes were based on best practices in the literature as well as on ethical standards to minimize numbers of animals for experiments and were dictated by the magnitude of experiment-to experiment variation. Statistical analysis was performed in GraphPad Prism 8.2.1 (GraphPad Software, USA) and Ingenuity Pathway Analysis (Qiagen). There was no explicit blinding or randomization.

For manuscripts utilizing custom algorithms or software that are central to the research but not yet described in published literature, software must be made available to editors/reviewers. We strongly encourage code deposition in a community repository (e.g. GitHub). See the Nature Research guidelines for submitting code & software for further information.

Data

Policy information about availability of data

All manuscripts must include a data availability statement. This statement should provide the following information, where applicable:

- Accession codes, unique identifiers, or web links for publicly available datasets
- A list of figures that have associated raw data
- A description of any restrictions on data availability

We state in the text "RNAseq data is available at the Gene Expression Omnibus (GEO) repository (GEO accession ID GSE126172; www.ncbi.nlm.nih.gov/geo). All other data that support the findings of this study are available from the corresponding author upon request."

Field-specific reporting

Please select the one below that is the best fit for your research. If you are not sure, read the appropriate sections before making your selection.

[x] Life sciences   [ ] Behavioural & social sciences   [ ] Ecological, evolutionary & environmental sciences

For a reference copy of the document with all sections, see nature.com/documents/nr-reporting-summary-flat.pdf

Life sciences study design

All studies must disclose on these points even when the disclosure is negative.

Sample size	Sample sizes were selected based on the previous examples of similar work in the literature e.g. Rouach et al. 2018, Pannasch et al. 2014
Data exclusions	No exclusions
Replication	Data was independently replicated using a minimum of three animals.
Randomization	No explicit randomisation was performed
Blinding	No explicit blinding was performed

Reporting for specific materials, systems and methods

We require information from authors about some types of materials, experimental systems and methods used in many studies. Here, indicate whether each material, system or method listed is relevant to your study. If you are not sure if a list item applies to your research, read the appropriate section before selecting a response.

Materials & experimental systems

n/a	Involved in the study	
[ ]	[x]	Antibodies
[ ]	[x]	Eukaryotic cell lines
[x]	[ ]	Palaeontology
[ ]	[x]	Animals and other organisms
[x]	[ ]	Human research participants
[x]	[ ]	Clinical data

Methods

n/a	Involved in the study	
[x]	[ ]	ChIP-seq
[x]	[ ]	Flow cytometry
[x]	[ ]	MRI-based neuroimaging

Antibodies

Antibodies used	anti-GAPDH [Abcam ab9484], anti-Cx43 [Sigma C6219], and anti-Cx30 [Invitrogen 71-2200], anti-HA antibody [Biolegend #901514], anti-GFP [abcam, ab13970], anti-cx43 [BD biosciences, 610061], anti-NeuN [cell signalling, D3S31], anti-s100b [ab41548]
Validation	All antibodies are commercially available and have been extensively validated by the manufacturer and in the literature.

Eukaryotic cell lines

Policy information about cell lines

Cell line source(s)	*State the source of each cell line used.*

Authentication	*Describe the authentication procedures for each cell line used OR declare that none of the cell lines used were authenticated.*
Mycoplasma contamination	*Confirm that all cell lines tested negative for mycoplasma contamination OR describe the results of the testing for mycoplasma contamination OR declare that the cell lines were not tested for mycoplasma contamination.*
Commonly misidentified lines (See ICLAC register)	*Name any commonly misidentified cell lines used in the study and provide a rationale for their use.*

Animals and other organisms

Policy information about studies involving animals; ARRIVE guidelines recommended for reporting animal research

Laboratory animals	C57BL/6J animals from The Jackson Laboratories were used. Animals were used between ages p25-45. Both sexes were used.
Wild animals	no wild animals
Field-collected samples	no field-collected samples
Ethics oversight	All animal protocols were approved by the University of Calgary Animal Care and Use Committee. Protocol numbers AC15-0053 and AC15-0133, AC17-0040

Note that full information on the approval of the study protocol must also be provided in the manuscript.
