# Supplementary material for: Stress gates an astrocytic energy reservoir to impair synaptic plasticity
Source: Nat Commun. 2020 Apr 24;11:2014. doi: 10.1038/s41467-020-15778-9 (PMC7181611; doi:10.1038/s41467-020-15778-9)
Supplement: Supplementary file 2 — Peer Review File [file 41467_2020_15778_MOESM2_ESM.pdf]

Editorial Note: This manuscript has been previously reviewed at another journal that is not operating a transparent peer review scheme. The manuscript was considered suitable for publication without further review at Nature Communications.
